# Scalable Route to Colloidal Ni_*x*_Co_3–*x*_S_4_ Nanoparticles with Low Dispersity Using Amino Acids

**DOI:** 10.1021/acsmaterialsau.3c00016

**Published:** 2023-07-10

**Authors:** Talisi E. Meyer, Kevin Zhijian Jiang, Ching Chun Peng, Quynh P. Sam, Minsoo Kang, Reilly P. Lynch, Jonathan L. Rowell, Judy Cha, Richard D. Robinson

**Affiliations:** † Department of Materials Science and Engineering, Cornell University, Ithaca, New York 14853, United States; ‡ Department of Chemistry and Chemical Biology, Cornell University, Ithaca, New York 14853, United States

**Keywords:** nanoparticles, ligands, spinel, ternary metal sulfides, surface chemistry

## Abstract

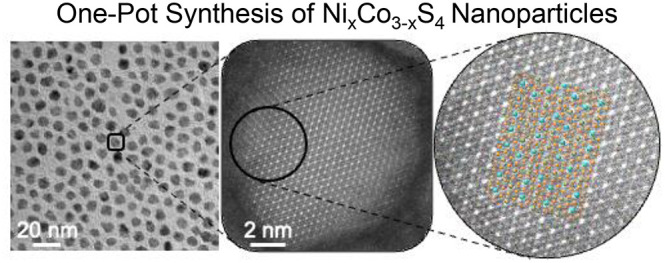

The thiospinel group of nickel cobalt sulfides (Ni_*x*_Co_3–*x*_S_4_) are promising materials for energy applications such as supercapacitors, fuel cells, and solar cells. Solution-processible nanoparticles of Ni_*x*_Co_3–*x*_S_4_ have advantages of low cost and fabrication of high-performance energy devices due to their high surface-to-volume ratio, which increases the electrochemically active surface area and shortens the ionic diffusion path. The current approaches to synthesize Ni_*x*_Co_3–*x*_S_4_ nanoparticles are often based on hydrothermal or solvothermal methods that are difficult to scale up safely and efficiently and that preclude monitoring the reaction through aliquots, making optimization of size and dispersity challenging, typically resulting in aggregated nanoparticles with polydisperse sizes. In this work, we report a scalable “heat-up” method to colloidally synthesize Ni_*x*_Co_3–*x*_S_4_ nanoparticles that are smaller than 15 nm in diameter with less than 15% in size dispersion, using two inexpensive, earth-abundant sulfur sources. Our method provides a reliable synthetic pathway to produce phase-pure, low-dispersity, gram-scale nanoparticles of ternary metal sulfides. This method enhances the current capabilities of Ni_*x*_Co_3–*x*_S_4_ nanoparticles to meet the performance demands to improve renewable energy technologies.

## 1 Introduction

In recent years, transition metal chalcogenides have received increased research attention due to their potential to be transformative to the field of energy conversion and storage.^1,2^ At present, the industrial space is dominated by metal oxides for applications in electrocatalysis, photocatalysis, supercapacitors, lithium-ion batteries, and sensing.^3^ Despite advancements by metal oxides for electrochemical applications, significant processing challenges have arisen due to issues such as demanding synthetic methods, difficulty in achieving efficient electrode contacts, and high manufacturing costs.^4^ Complex metal sulfides (ternary, quaternary, etc.) are emerging materials that have the potential to overcome the challenges faced by metal oxides due to their improved electrochemical activity, mechanical and thermal stability, as well as low-cost processing compared to metal oxides and monometallic sulfides.^5,6^ The thiospinel group of nickel cobalt sulfides (Ni_*x*_Co_3–*x*_S_4_), specifically, have higher conductivities,^7^ smaller optical energy band gaps,^8^ and better ductility^9^ than their counterpart, nickel cobalt oxides. For electrochemical applications, Ni_*x*_Co_3–*x*_S_4_ performs better than monometallic sulfides (NiS_*x*_ and CoS_*x*_) due to the mixed oxidation states of Ni and Co in Ni_*x*_Co_3–*x*_S_4_.^8,10^ While various morphologies have been used,^11−18^ a fundamental structure–morphology–property connection has been difficult to establish, limiting advances to empirically derived studies. The ideal testbed is to examine materials with uniform shapes and sizes, with a simple and replicable geometry. Sphere-like nanoparticles with monodisperse sizes (statistical size distribution below 20%) would enable researchers to assess the underlying chemical and physical mechanisms and link them to device performance properties.

The synthesis techniques commonly employed, including solvothermal and hydrothermal methods,^19−21^ pose significant obstacles for the industrial utilization of NiCo_2_S_4_ due to issues like oversized particles, uneven particle distribution, the presence of single-metal sulfide impurities, and the inherent difficulty in scaling up these methods. For instance, Yu et al. reported monodisperse hollow Ni-Co-S nanoprisms through the solvothermal method, but the materials required a post-synthetic annealing step for crystallization and their size was on the order of microns.^22^ Similarly, Rajesh et al. utilized the hydrothermal method to produce large (greater than 5 microns in size), polydisperse rambutan-like cobalt nickel sulfide.^23^ The amorphous nickel cobalt sulfide showed a specific capacitance of 895 F/g at 1 A/g, while the flower-like cobalt sulfide showed a specific capacitance of 1102.22 F/g at the same current density, highlighting the performance inhibition caused by non-spherical shapes and large sizes within particles. In contrast, Chen et al. utilized colloidal hot injection techniques to generate nickel cobalt sulfide quantum dots, reportedly achieving a size distribution below 15% according to their findings.^24^ However, it is challenging to verify this value solely based on the transmission electron microscopy (TEM) images presented. Apart from these reports, there is little evidence available of scalable synthetic methods to produce monodisperse, nanometer-sized particles. Currently, the major limitation of metal sulfide nanostructures in widespread industrial applications is the lack of production methods that are low-cost, efficient, reproducible, and simple.^8,25^ Thus, there is need for an inexpensive, scalable technique to create particles with a sphere-like nature that are uniform in size.

In this work, we introduce the scalable synthesis of phase pure, low dispersity, sphere-like nickel cobalt sulfide nanoparticles through a reliable synthetic pathway. Using low-cost sulfur precursors, including the amino acid l-cysteine ethyl ester hydrochloride (LCEE) and elemental sulfur, we have developed a tunable system with compositional control, yielding gram-scale quantities, which is applicable to multiple ternary metal sulfide systems. We have uncovered a reaction mechanism utilizing amino acids as a sulfur and ligand source as well as elemental sulfur to act as an etchant^26^ to improve the size and shape of ternary metal sulfide nanoparticles.^27^ Our synthesis employs two surfactants to act as stabilizing agents to control the size and shape of nanoparticles and provide surface protection.^28−30^ Our system has been evaluated for practical use in energy storage applications through analysis of our active material on nickel foam electrodes in a three-electrode cell. These Ni_0.8_Co_2.2_S_4_ electrodes deliver a high energy density of 37.6 Wh/kg at a power density of 1867.7 W/kg, in line with current literature.^12,24,31−34^

## 2 Experimental Section

### 2.1 Materials

Nickel(II) acetylacetonate (Ni(acac)_2_, 95%), cobalt(II) acetylacetonate (Co(acac)_2_, 99%), sulfur (S, 99%), oleylamine (OLAM, 98%), oleic acid (OLAC, 90%), Nafion (5 wt %), and nitric acid (HNO_3_, 70% redistilled) were purchased from Sigma-Aldrich. Hydrochloric acid (HCl, 37%) was purchased from MilliporeSigma. Potassium hydroxide (KOH) was purchased form VWR Chemicals. LCEE (98%) was purchased from Thermo Scientific. Hexane, acetone, and dichloromethane (certified ACS) were purchased from Fisher Chemical. Ethanol (200 proof) was purchased from Koptec. Super P carbon black powder was purchased from MSE Supplies. All chemicals were used as received.

### 2.2 Synthesis of NiCo_2_S_4_ Nanoparticles

In a 100 mL three-neck round-bottom flask, Ni(acac)_2_ (113 mg, 0.44 mmol), Co(acac)_2_ (226 mg, 0.88 mmol), LCEE (326 mg, containing 1.76 mmol of S), and elemental sulfur (7 mg, 0.22 mmol) were dissolved in OLAM (32 mL) and OLAC (8 mL). A magnetic stir bar was placed in the flask to constantly stir the reaction mixture throughout the entire reaction. The solution was degassed under vacuum at 110 °C for 1 h to remove low boiling point impurities. Then, the reaction mixture was heated to 170 °C under nitrogen and held at 170 °C for 3 h. At the end of the reaction, the reaction mixture was quenched to room temperature using a water bath and either transferred into centrifuge tubes for further washing or kept in the three-neck flask for distillation. To achieve other stoichiometries, the metal cation ratios were varied. To test application to other ternary metal syntheses, 0.44 mmol of Ni(acac)_2_ or Co(acac)_2_ was dissolved with 0.88 mmol M(acac)_2_, where M represents alternative transition metals. All other parameters were maintained.

### 2.3 Post-Synthesis Washing

The quenched reaction mixture was transferred equally into two centrifuge tubes (20 mL reaction solution each). Ethanol (30 mL) was added to each tube and centrifuged at 4400 rpm for 5 min to flocculate the nanoparticles. Then, the precipitate in each tube was re-dispersed in hexane (15 mL), precipitated with acetone (30 mL), and recovered by centrifuging at 4400 rpm for 5 min. After discarding the supernatant, the precipitates in each tube were once again re-dispersed in hexane (15 mL), mixed with acetone (30 mL), and recovered by centrifuging at 4400 rpm for 5 min, and the final product was allowed to dry overnight in a fume hood.

### 2.4 Vacuum Distillation

Distilled particles omitted the post-synthesis washing step. The synthesized nanoparticles were kept overnight within a sealed three-neck round bottom flask in a 4 °C fridge. Prior to distillation, the flask was removed from the fridge and allowed to reach room temperature under nitrogen. Under vacuum, the synthesized nanoparticles were then slowly heated from room temperature until condensation was observed in the column and collected in single neck round bottom flasks cooled by an acetone and dry ice bath. This temperature was maintained or raised no more than 5 °C until all material was removed. Multiple fractions were collected from each synthesis using this method including at 145–150 °C, 160–165 °C, and above 170 °C.

### 2.5 Characterization

#### 2.5.1 TEM and High-Angle Annular Dark-Field Scanning TEM

TEM images were acquired using an FEI Tecnai 12 BioTwin TEM with a LaB_6_ source at 120 kV accelerating voltage. High-angle annular dark-field scanning TEM (HAADF-STEM) images were acquired using an aberration-corrected Thermo Fisher Spectra 300 at 300 kV accelerating voltage.

#### 2.5.2 Powder X-ray Diffraction

Primary X-ray diffraction (XRD) patterns were collected using the Bruker D8 Advance General Area Detector Diffraction System with a 1.6 kW Co-kα X-ray source to minimize the background fluorescence signal. Additional XRD patterns were obtained using a Bruker D8 Advance ECO powder diffractometer with a 1.2 kW Cu-kα X-ray source and a silicon strip detector with the discriminator set to 0.182–0.220 V. The acquired XRD pattern was matched to a reference pattern obtained on FIZ Karlsruhe Inorganic Crystal Structure Database (ICSD). The peak profile of the strongest reflection plane was baseline-corrected and fitted to a pseudo-Voigt function to determine its full width at half maximum, which was then used in the Scherrer equation to calculate the average crystallite size of nanoparticles in the sample.

#### 2.5.3 Inductively Coupled Plasma Optical Emission Spectroscopy

Inductively coupled plasma optical emission spectrometry (ICP-OES) data were measured on a Thermo iCAP 6500 series equipped with an argon torch at the Cornell Nutrient Analysis Laboratory. Samples were dissolved in 1 mL of HNO_3_ and diluted in 29 mL of deionized water for analysis.

#### 2.5.4 Nuclear Magnetic Resonance (^1^H NMR and ^13^C NMR) Spectroscopy

All NMR spectra were collected using the 500 MHz Bruker AVIII with BBO Prodigy cryoprobe at Cornell NMR and Chemistry Mass Spectrometry Facilities. NMR samples were prepared in chloroform-d. Quantitative ^1^H spectra were collected over 4 scans with 30 s relaxation delay and 90° excitation pulse at room temperature, and ^13^C NMR spectra were collected with ^1^H decoupling and 128 scans over 10 min.

#### 2.5.5 Fourier Transform Infrared Spectroscopy

Fourier transform infrared spectroscopy (FTIR) spectra were obtained using a Bruker Vertex V80V vacuum FTIR system collected over 64 scans from 4000 to 700 cm^–1^ with 4 cm^–1^ scan resolution. FTIR spectra of nanoparticles were collected under transmission mode, where <2 mg nanoparticles were mixed with ∼250 mg KBr powder and vacuum-pressed into disks before the measurement. FTIR spectra of all other samples were collected under the attenuated total reflectance mode.

#### 2.5.6 Thermogravimetric Analysis

Thermogravimetric analysis (TGA) measurements were performed using TA Instruments 5500 thermogravimetric analyzer. TGA curves of precursors and synthesized nanoparticles were collected by placing a 5–10 mg sample into a tared high temperature Pt pan and heating to 400 °C with a consistent ramp rate of 10 °C per min under constant nitrogen flow. TGA curves mimicking the full heat-up reaction were collected by taking an aliquot after the vacuum step of synthesis and heating it from room temperature to 170 °C with a ramp rate of 5 °C per min under nitrogen. Isothermal conditions were maintained at 170 °C for 1 h before heating to 700 °C at a ramp rate of 10 °C per min.

#### 2.5.7 X-ray Photoelectron Spectroscopy

X-ray photoelectron spectroscopy (XPS) measurements were performed using a Scienta Omicron ESCA 2SR spectrometer with a monochromatized Al Kα excitation source. Photoelectrons were collected from 1.1 mm diameter analysis spots. Samples were prepared by depositing nanoparticles onto a conductive carbon tape on a clean Si wafer. Three scans of random locations were surveyed for nickel and cobalt to ensure representative scans and confirm sample homogeneity. Photoelectrons were collected at a 90° emission angle with a source-to-analyzer angle of 54.7°. A hemispherical analyzer determined electron kinetic energy, using a pass energy of 200 eV for wide/survey scans, and 50 eV for high resolution scans. A flood gun was used for charge neutralization of non-conductive sample surfaces. All binding energies were calibrated using the C 1s peak. A Shirley background was used to subtract the background for Ni 2p and Co 2p peaks. The extracted spectra were fitted with a 70%/30% Gaussian/Lorentzian line shape.

## 3 Electrochemical Measurements

### 3.1 Electrode Fabrication

Super P carbon black (5 mg) was first suspended into chloroform (10 mL) and sonicated for 1 h. In a separate container, Ni_*x*_Co_3–*x*_S_4_ nanoparticles (25 mg) were suspended in chloroform (10 mL). The Ni_*x*_Co_3–*x*_S_4_ nanoparticle solution was then added slowly into the carbon black, and the suspension was dried at 45 °C while stirring under nitrogen flow. Ligands were removed from the nanoparticle surface by annealing the dry mixture of Super P carbon black powder and Ni_*x*_Co_3–*x*_S_4_ nanoparticles with a weight ratio of 1:5 at 250 °C under nitrogen for 10 h followed by air cooling to room temperature. After heat treatment, the powder was mixed with Nafion with a weight ratio of 9:1, and ethanol (1 mL) was also added as a solvent to create a slurry. The slurry was then drop-cast onto a nickel foam substrate, and the composite electrode was allowed to dry overnight at 80 °C under nitrogen. The geometric surface area of all electrodes was held constant at 2 cm^2^, and calculations were conducted from the active mass loading, excluding the mass of the binder, conductive additive, and the substrate used. The weight loss due to the ligand removal was considered, with around 23% loss confirmed by TGA. The average weight of the active material of Ni_*x*_Co_3–*x*_S_4_ analyzed was 1.5 mg.

### 3.2 Electrochemical Testing

The electrochemical performance of the nanoparticle-coated electrode was investigated using a three-electrode electrochemical cell. The working electrode was the nanoparticle-coated electrode. The reference electrode was an Ag/AgCl glass electrode in a saturated KCl solution. The counter electrode was a Pt wire, and the electrolyte was 6 M KOH solution. All electrodes were connected to a BioLogic VMP3 potentiostat. The electrolyte solution was purged for several hours before measurements to minimize the influence of dissolved oxygen on the electrochemical system. The electrochemical performance of the nanoparticle-coated electrode was characterized using cyclic voltammetry (CV) and galvanostatic charge–discharge (GCD) cycling. For CV, the electrode was cycled between 0 and 0.5 V at various voltage scan rates, ranging from 2 to 60 mV/s. For GCD, the electrode was cycled between 0 and 0.4 V at various current densities, ranging from 1 to 50 A/g.

## 4 Results and Discussion

### 4.1 Synthesis Result

**Scheme 1:**
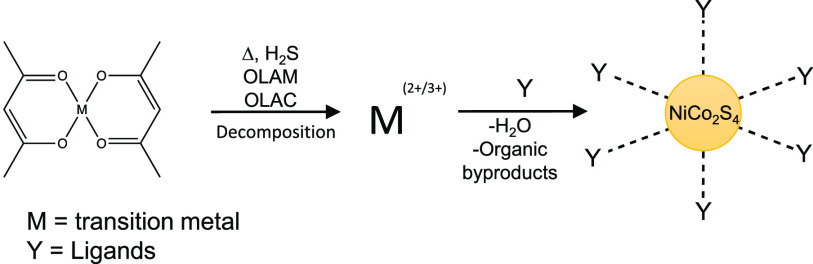
Proposed Reaction Mechanism General reaction mechanism for the formation of Ni_x_Co_3–x_S_4_ nanoparticles in OLAM and OLAC. H_2_S (confirmed through hydrogen sulfide test strips) from LCEE or sulfur reacts with a transition metal (M) acetylacetonate, which decomposes with increasing temperatures. Ligands (Y) on the nanoparticle surface are dependent on the sulfur source(s) used during an otherwise typical Ni_0.80_Co_2.20_S_4_ synthesis.

Ni_*x*_Co_3–*x*_S_4_ nanoparticles were synthesized with a simple one-pot, “heat-up” approach based on the thermal decomposition of metal–organic precursors in a concentrated ligand environment of 100 mol of ligands per 1 mol Ni_*x*_Co_3–*x*_S_4_. Ni(acac)_2_ and Co(acac)_2_, which were chosen because of their similar chemical compositions and thermal stability, which provides a similar decomposition rate and promotes equal incorporation of Ni and Co cations into the nascent nanoparticles. LCEE, an amino acid commonly used in food processing and pharmaceuticals,^35,36^ was employed as a safe and low-cost sulfur precursor and was used as the main sulfur source in our synthesis. Elemental sulfur was also used in conjunction with LCEE at a much lower quantity (8 times more S from the LCEE than from the elemental sulfur precursor) to improve the shape and size dispersion of the nanoparticles. Hereafter, a “Ni_0.8_Co_2.2_S_4_ synthesis” will refer to conditions with a molar ratio of 0.44 mmol Ni(acac)_2_, 0.88 mmol Co(acac)_2_, 1.76 mmol LCEE, and 0.22 mmol sulfur (1:2:4:0.5) dissolved in OLAM and OLAC (Scheme 1). This mixture was first heated to 110 °C at a ramp rate of 5 °C per min under vacuum, then heated under N_2_ to a soak temperature of 170 °C at the same ramp rate, and allowed to soak at this temperature for 3 h. The experimental design was optimized beginning with parameters shown to be most significant to final product size/size dispersion, shape, and phase.^37^ Parameters such as the soak time and temperature were optimized through a time (Figure S1) and temperature study (Figure S2) with particle sizes ranging between 7.5 and 10.1 nm. As will be discussed, the temperature range of the reaction was determined through TGA analysis of neat precursors. In a typical Ni_0.8_Co_2.2_S_4_ synthesis, OLAM acts as the primary ligand either neat or through interactions with LCEE and sulfur. Interactions of OLAM with sulfur also form an etchant species,^26^ which enables size and shape control of nanoparticles, while OLAC acts as a stabilizing agent limiting the growth of nanoparticles^28^ and improving the uniformity of nanoparticle size and shape. By maintaining molar ratios of all precursors, this reaction can be scaled to 10 times the standard Ni_0.8_Co_2.2_S_4_ synthesis conditions, yielding over 1.59 g of nanoparticles consisting of approximately 23% organic byproducts and an average size of 9.2 nm ± 12.8% (Figure S3).

**Figure 1:**
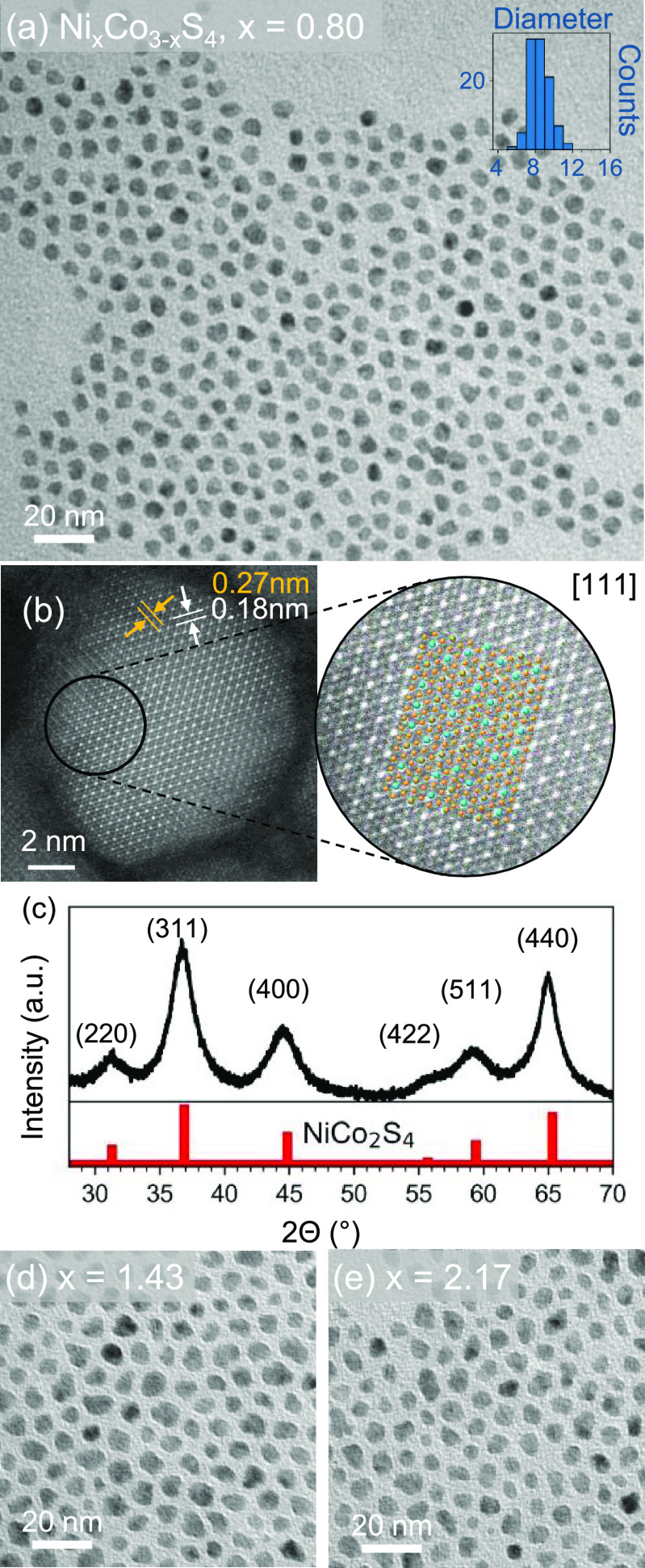
Synthetic tunability of monodisperse Ni_*x*_Co_3–*x*_S_4_ nanoparticles. (a) TEM images of Ni_0.8_Co_2.2_S_4_ synthesis show sphere-like nanoparticles with (inset) statistical size distribution of ±12.4%. (b) HAADF-STEM image of a single crystalline *x* = 0.80 nanoparticle along the [111] axis corresponding to the spinel phase. Nickel is represented by cyan and cobalt is represented by orange. (c) XRD pattern further supports spinel phase. Red bars correspond to ICSD standard #624472 for NiCo_2_S_4_. (d,e) TEM images show highly uniform particle morphology and atomic structure for altered Ni content (*x* = 1.43, and 2.17, respectively).

TEM images of a Ni_0.8_Co_2.2_S_4_ synthesis show sphere-like, monodisperse nanoparticles, with an average size of 10.1 nm ± 12.4% (Figure 1a). HAADF-STEM images indicate that a mixture of single crystalline (Figure 1b) and poly-crystalline nanoparticles (Figure S4) is present. The single crystalline nanoparticles possess clearly defined lattice fringes and an atomic structure of the spinel phase. The measured lattice spacings of 0.27 and 0.18 nm correspond to the (311) and (440) planes, respectively, indicating a [111] zone axis consistent with literature.^38^

By varying the precursor ratios of nickel and cobalt in a synthesis, compositional control of a range of stoichiometries was also achieved. Specifically, compositions of Ni_*x*_Co_3–*x*_S_4_ nanoparticles determined by ICP-OES (Figure S5g) include *x* = 0.80 (Figure S5a, 1Ni:2Co), 1.43 (Figures 1d and S5b, 1.5Ni:1.5Co), and 2.17 (Figures 1e and S5c, 2Ni:1Co). The synthesis method using 0.44 mmol of Ni(acac)_2_/Co(acac)_2_ and an additional 0.88 mmol of a 2+ transition metal acetylacetonate is shown to be applicable to other ternary thiospinels, producing nanoparticles with a statistical size distribution below 20%. Nickel iron sulfide (NiFe_2_S_4_) and cobalt manganese sulfide (Co_0.2_Mn_0.8_S) were synthesized and evaluated through TEM (Figure S6a,b) and XRD (Figure S6c,d). The nickel iron sulfide nanoparticles have an average size of 35.6 nm ± 13.6% (Figure S6a, inset), and cobalt manganese sulfide nanoparticles have an average size of 19.8 nm ± 11.5%, (Figure S6b inset). Excess cobalt within the system reacts with excess sulfur forming Co_3_S_4_ as well.

XRD indicates that the products of a Ni_0.8_Co_2.2_S_4_ synthesis are a phase-pure spinel-type structure. All XRD patterns have strong reflections matching the angle and intensity of the reflecting planes for the thiospinel NiCo_2_S_4_ phase, with no distinguishable impurity reflections (Figure 1c). Reflection angles for this synthesis appear at 26.7°, 31.3°, 37.95°, 47.1°, 49.9°, and 54.9° corresponding to the (220), (311), (400), (422), (511), and (440) diffraction planes of NiCo_2_S_4_,^12,24,31^ respectively (ICSD standard #624472). Stoichiometries beyond the ratios described in a Ni_0.8_Co_2.2_S_4_ synthesis (Figure S5d–f) and other amino acids (Figure S7c) were also determined through XRD. By fitting a pseudo-Voigt function to the strongest and most symmetrical diffraction peak (Figure S9), the mean crystallite size of various reactions was calculated to be 4.58 nm (*x* = 0.80), 4.34 nm (*x* = 1.43), and 3.87 nm (*x* = 2.17), all within the range of values determined by TEM.

**Figure 2:**
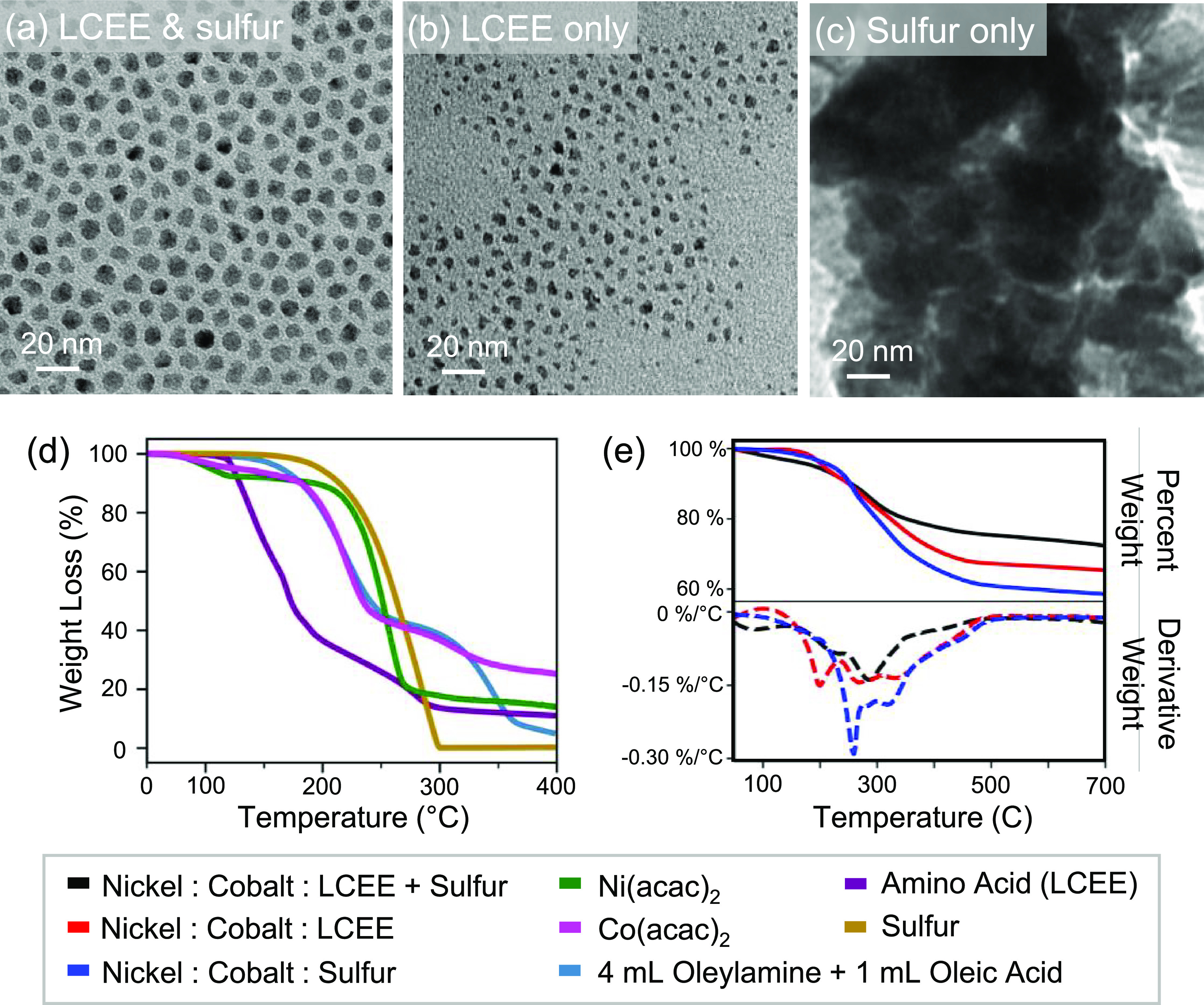
Comparison of Ni_0.80_Co_2.20_S_4_ nanoparticles synthesized following a typical synthesis procedure with 1.96 mmols of sulfur precursors altered as follows: both LCEE and sulfur, LCEE only, and sulfur only. All other parameters of a typical Ni_0.80_Co_2.20_S_4_ synthesis were maintained throughout the three syntheses. (a–c) TEM images of LCEE and sulfur, LCEE only, and sulfur only reactions. (d) TGA curves of neat precursors: Ni(acac)_2_ and Co(acac)_2_, LCEE, elemental sulfur, 4 mL of OLAM +1 mL OLAC. All samples were analyzed from 50 to 400 °C and heated at a rate of 10 °C per min under constant nitrogen flow. LCEE and the metal precursors are shown to begin decomposing at the lowest temperatures (below 150 °C) followed by OLAM and OLAC (∼160 °C) and sulfur (∼180 °C). (e) Post-synthetic TGA analysis of the three nanoparticle products after washing shows the relative organic loading. Reactions employing one sulfur source show a higher total percent weight loss, which indicated a larger organic loading compared to syntheses utilizing both LCEE and elemental sulfur.

Through TEM analysis of the particles’ size and shape, the optimal ratio of the two-sulfur source system was found to be 4 moles of LCEE to 0.5 moles of sulfur (along with 1 mole Ni(acac)_2_ and 2 moles Co(acac)_2_). As evidenced through TEM, using a molar quantity of 1.96 mmol LCEE (equal to the total amount of sulfur added from LCEE and elemental sulfur to a typical Ni_0.8_Co_2.2_S_4_ synthesis), while maintaining all other parameters the same, a Ni_0.8_Co_2.2_S_4_ synthesis will result in nanoparticles without a uniform morphology and with a higher size distribution compared to the two-sulfur method (Figure 2b). Equally, when only elemental sulfur is used as the sulfur source, the synthesized particles are large and agglomerated (Figure 2c). By combining LCEE and sulfur at an 8 to 1 molar ratio (1.76 mmol LCEE + 0.22 mmol sulfur), the nanoparticles possess the increased growth exhibited by sulfur alone but are monodisperse and sphere-like (Figure 2a) due to the shape control offered by LCEE. Additionally, this reaction method is replicable with the amino acid l-cysteine in place of LCEE for a typical Ni_0.8_Co_2.2_S_4_ synthesis, forming monodisperse particles (Figure S7a) with an average size of 10.2 nm ± 17.9% (Figure S7b).

Previous work has shown that OLAM and OLAC may both act as reducing agents as well as surface protection and can impact the growth of nanoparticles affecting their size and shape.^28,29,39^ An optimal ratio of 4 mL of OLAM to 1 mL of OLAC with a total of 40 mL of surfactants within a Ni_0.8_Co_2.2_S_4_ synthesis was found to give monodisperse, sphere-like particles. TEM images of a Ni_0.8_Co_2.2_S_4_ synthesis using 40 mL of surfactants but at varied volume ratios exhibited mixed morphologies in OLAC only (Figure S8a), agglomerated particles with high OLAM ratios (Figure S8b,c), and polydisperse particles with equal amounts of OLAM and OLAC (Figure S8d). Utilizing a volume ratio of 4 mL of OLAM to 1 mL of OLAC results in particles with the uniform morphology and low size dispersion (Figure 1a).

### 4.2 Surface Characterization

Surface characterization reveals that the likely nucleation pathway is through the decomposition of the LCEE and metal precursors above 150 °C followed by a solution sulfidation process.^12^ Through TGA studies of the precursors, the LCEE decomposes initially and rapidly around 150 °C, before the other precursors including elemental sulfur (Figure 2d). Replications of a Ni_0.8_Co_2.2_S_4_ synthesis through TGA using only one sulfur source (either LCEE or sulfur) compared to both sulfur sources show that the decomposition pathway for the combined LCEE with sulfur synthesis resembles an LCEE-only synthesis, indicating that the elemental sulfur does not alter the reaction of the LCEE (Figure S10); this result also supports the idea of LCEE forming the initial nuclei. Previous work found that l-cysteine decomposes above 150 °C into three species: H_*2*_S, −CNH_2_, and −COOR,^40^ where the R represents −CH_2_CH_3_ if LCEE is used or −H if l-cysteine is used. We therefore conclude that the H_2_S from the amino acid is creating the nuclei through the sulfidation process with the metals.

Post-synthetic TGA and derivative TGA curves of Ni_0.80_Co_2.20_S_4_ nanoparticle products synthesized with either LCEE only, sulfur only, or LCEE + sulfur indicate that the ligand density on the nanoparticles is dependent on the sulfur precursor(s) used (Figure 2e). Specifically, derivative TGA curves (Figure 2e bottom curve) show that the surface ligands of nanoparticles synthesized with only LCEE experience a rapid weight loss at nearly 100 degrees lower (249 °C) compared to reactions utilizing elemental sulfur alone or in tandem with LCEE, indicating a much lower decomposition temperature. Initially, the reaction using only LCEE decomposes quicker than other reactions; however, above 200 °C, the reaction utilizing elemental sulfur as the sole sulfur source experiences a larger total decrease in weight percent, resulting in a 19% weight loss between 200 and 300 °C. Reactions using LCEE, with and without elemental sulfur, exhibit a weight loss of 10 and 15%, respectively, between 200 and 300 °C. The second peak between 300 and 450 °C indicates the complete decomposition of ligands on the nanoparticle surface. By 500 °C, the sulfur-only reaction experiences a total weight loss of 40% and the LCEE-only reaction experiences a total weight loss of 33% corresponding to the contribution of organics remaining after the washing procedure. The reaction with both LCEE and sulfur experiences the least rapid decrease in initial mass overall and exhibits a weight loss of 25% of the initial mass by 500 °C. This result further supports the advantage of utilizing the two-sulfur synthesis. This decomposition at approximately 300 °C corresponds to the expected decomposition of the surfactants used in the reaction (see Figure 2d), implying the presence of one or both surfactants on the nanoparticle surface. TGA analysis of the scaled synthesis shows a total weight loss of 23%, indicating that approximately 0.36 g of the 1.56 g total is contributed by organic byproducts and ligands and the final yield of nanoparticles is 1.20 g.

**Figure 3:**
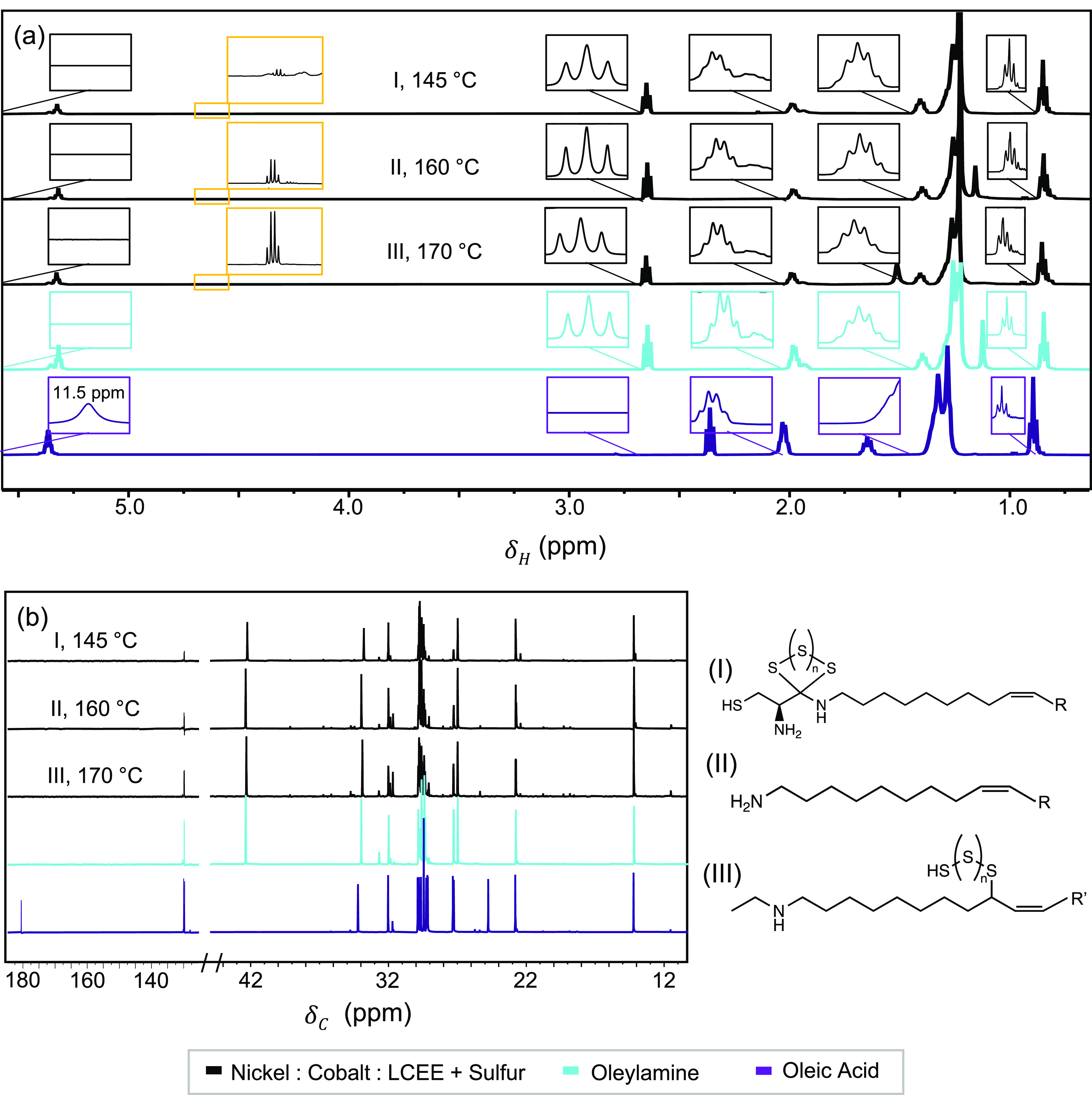
Analysis of the surface morphology of Ni_0.80_Co_2.20_S_4_ nanoparticles through (a) ^1^H NMR and (b) ^13^C NMR spectra of distillations fractions (removed at the given temperatures) compared to neat OLAM and OLAC. Results indicate that OLAM, not OLAC, is on the surface of the nanoparticles in the form of three distinct ligands: (I) a thioacetal formed by LCEE, sulfur, and OLAM, (II) neat OLAM, and(III) OLAM with a sulfur bridge.

^1^H NMR and ^13^C NMR spectra of distillation fractions of the synthesized Ni_0.80_Co_2.20_S_4_ nanoparticles indicate that OLAM and not OLAC is the key surfactant on the nanoparticle surface, playing a role in the formation of a thioacetal-LCEE-OLAM complex, an ethyl-OLAM species, and acting as a ligand itself. Distillation fractions were taken of each synthesis to account for the line broadening associated with surface bound ligands in colloidal nanoparticles^41^ such as nickel cobalt sulfide (Figure S11). From ^1^H NMR (Figure 3a), we find variations in the splitting pattern and position of peaks between neat OLAM and OLAC, and the peaks present in distillation fractions of the Ni_0.8_Co_2.2_S_4_ nanoparticles match those of OLAM. Specifically, within our surfactants the carbon chain is at 0.90 ppm in OLAC, and it shifts upfield to 0.88 ppm for the neat OLAM and nanoparticle spectra (Figure 3a light blue and black plot, respectively). Also, in OLAC, the hydrogen located adjacent to the carboxylic acid is indicated by a triplet at 2.34 ppm (Figure 3a purple plot).^42^ In OLAM, the hydrogen adjacent to the amine group is indicated by a triplet at 2.65 ppm (Figure 3a light blue plot).^43,44^ The hydrogen alongside the double bond of OLAM and OLAC is indicated by a multiplet at 1.99 ppm and a quartet at 2.04 ppm, respectively.^44,45^ In all Ni_0.80_Co_2.20_S_4_ distillation fractions, the peak present at 1.97 ppm corresponds in both shape and position to that of OLAM. Other evidence for OLAM on the surface includes the amine peak at 1.17 ppm and the carbon chain peak at 1.41 ppm, both of which occur in OLAM and the NPs but not in neat OLAC. In OLAC, the alcohol corresponds to a peak at 11.5 ppm (Figure 3a purple plot).^46^ This peak is absent in all distillation fractions. From ^13^C NMR (Figure 3b), there are two specific peaks that separate OLAM and OLAC. OLAC presents a carboxylic acid indicated peak at 180 ppm (Figure 3b purple plot).^47^ This peak is not present in the nanoparticle fractions (Figure S12). OLAM presents an amine peak at 42 ppm^27^ that is distinctly present in all nanoparticle distillation fractions (Figure 3b). The observed peak differences indicate that OLAM, not OLAC, is present on our nanoparticle surface, both following reaction with the sulfur sources and acting as a ligand itself.

**Figure 4:**
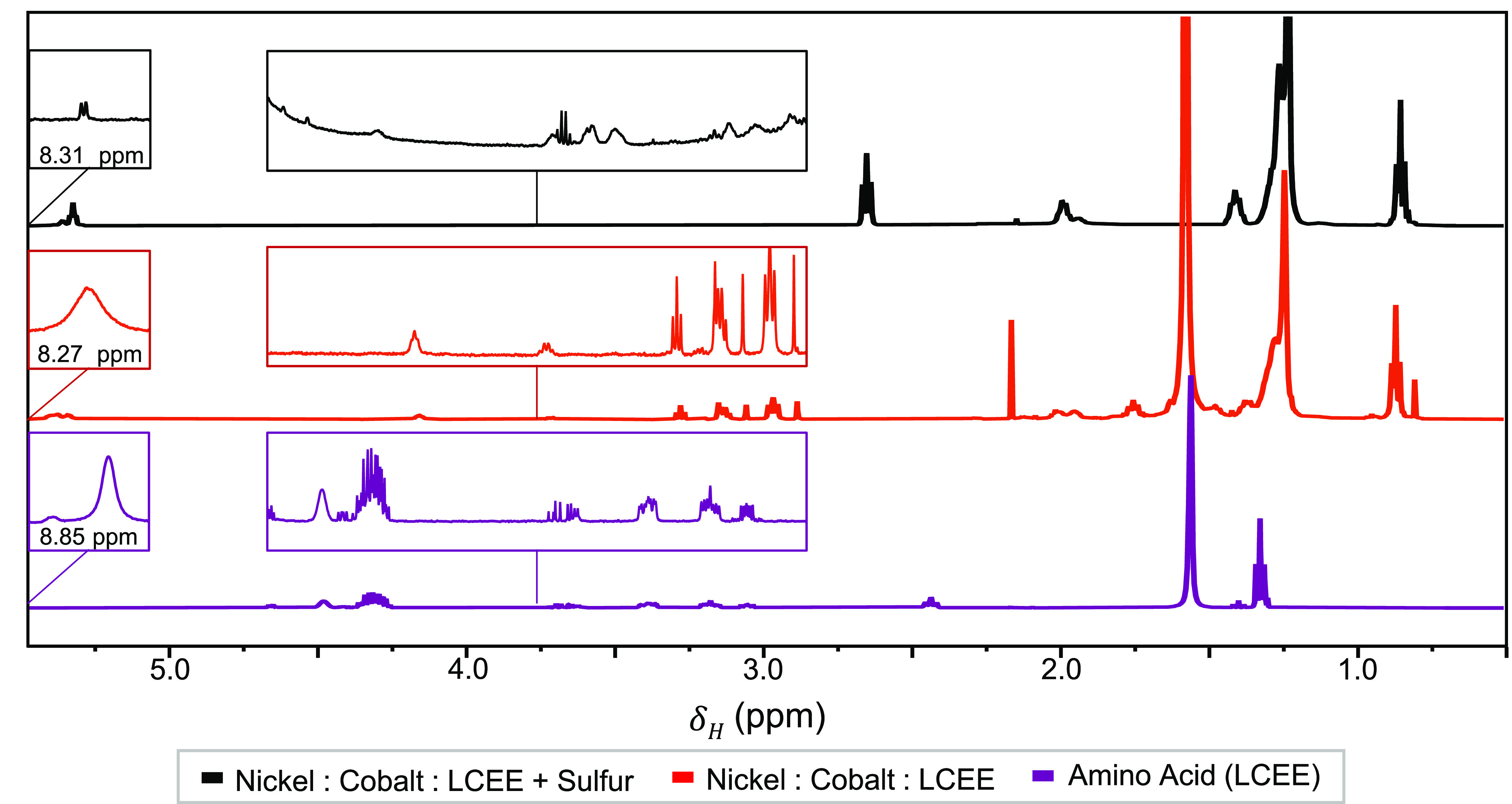
^1^H NMR spectra show characteristic peaks of LCEE in distillations fractions of syntheses employing LCEE (purple), with (black) and without (red) elemental sulfur, removed at 145 °C. Proposed reaction mechanisms leading to the ligands found on the nanoparticle surface of the lower distillation fractions. (Scheme 2) l-Cysteine reacts with OLAM in a condensation reaction to form an amide. Excess OLAM can react with the −OR''^–^ leaving group to form a secondary amine when R'' = CH_2_CH_3_.

**Scheme 2:**
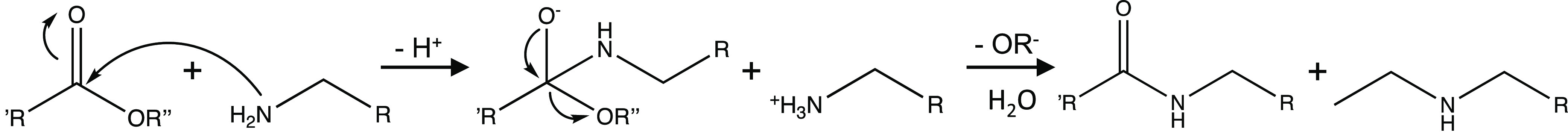
Proposed Mechanism—-Amide Formation from LCEE and OLAM

**Scheme 3:**
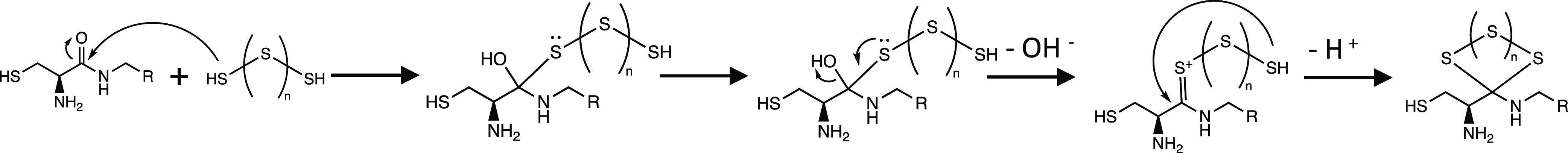
Proposed Mechanism—Thioacetal Reaction of LCEE-OLAM amide with Sulfur

Distillation fractions removed at the lowest temperature (145 °C) indicate that an amide formed by OLAM and LCEE reacts with excess elemental sulfur to form a thioacetal group in place of the oxygen. ^1^H NMR analysis of fractions distilled at 145 °C (Figure 4) finds that syntheses with only LCEE result in a surface that is rich in an amide, formed by the condensation reaction of LCEE and OLAM (Scheme 2). The presence of the secondary amide within the LCEE only reaction is indicated by the singlet at 8.27 ppm (Figure 4 red plot) similar to the peak of neat LCEE at 8.85 ppm (Figure 4 purple plot), which is assigned to the proton directly bound to the nitrogen.^48^ Peaks at 3.14 and 2.97 ppm are assigned to protons de-shielded by adjacent thiol and amine groups, respectively, from the excess non-decomposed LCEE precursor (Figure 4 red plot).^48^ In fractions of a typical Ni_0.8_Co_2.2_S_4_ synthesis distilled at 145 °C, the unique peaks seen at 2.97 ppm and 3.14 in an LCEE-only reaction as well as a doublet at 8.31 ppm (in place of the singlet seen in LCEE due to the basic environment^48^) are present at much smaller intensities (Figure 3a, black plot). Instead, in a two-sulfur synthesis, the bonds associated with OLAM dominate. Further analysis through ^13^C NMR provides evidence that elemental sulfur may react with the oxygen present on the carbonyl group of the LCEE-OLAM amide forming a thioacetal (Figure 4, Scheme 3), as indicated by a peak at 77.36 ppm (Figure S12b, black plot). This peak is in addition to the three major triplet peaks corresponding to CHCl_3_ in the 77 ppm range and is not seen in ^13^C NMR analysis of other distillation fractions. The evidence of this amide reaction with sulfur is only observable in scaled concentrations of a Ni_0.80_Co_2.20_S_4_ nanoparticle synthesis, suggesting that while the amide formed by LCEE and OLAM is the predominant ligand in reactions employing only LCEE (Figure 4, red plot), it is not the dominant ligand when sulfur is incorporated as well. Other peaks seen by ^13^C NMR correspond to the double bond (130.47 to 129.95 ppm), the −CH_2_ groups of carbon chain (33.82 to 22.45 ppm), and the amine group (42.40 ppm) of OLAM (Figure S12). In distillations using elemental sulfur as the only sulfur source, no material is removed before 155 °C, further confirming the presence of LCEE in the distillation fractions of syntheses when LCEE is used. Based on this evidence, it is apparent that a minor fraction of ligands on the nanoparticle surface are formed by a reaction of LCEE, OLAM, and elemental sulfur resulting in the formation of a thioacetal species.

**Figure 5:**
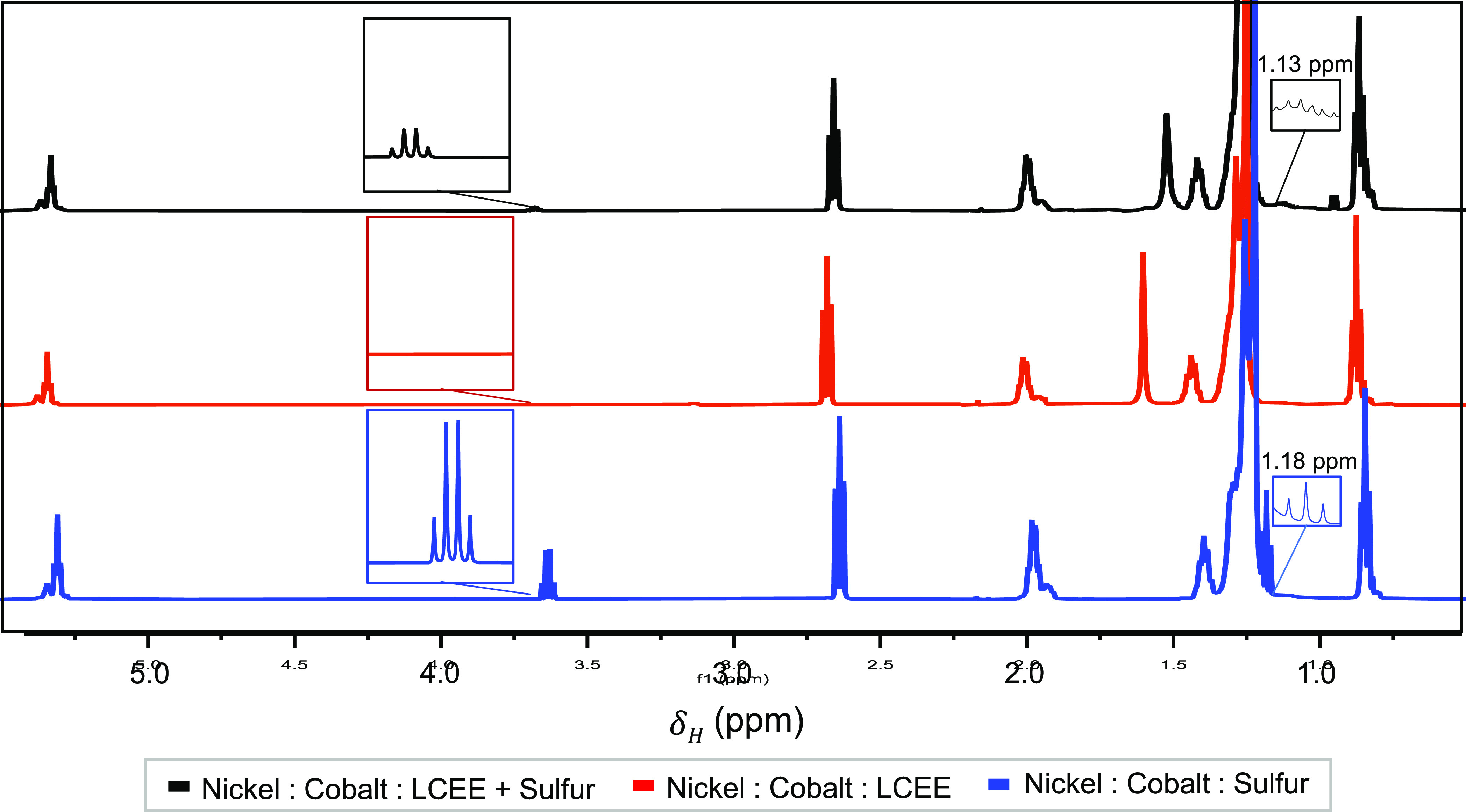
^1^H NMR spectra show the influence of elemental sulfur within the nickel cobalt sulfide synthesis employing two sulfur precursors. Proposed reaction mechanisms leading to the ligands found on the nanoparticle surface and an etchant in a Ni_0.8_Co_2.2_S_4_ synthesis. (Scheme 4) Elemental sulfur, following thermal decomposition, can interact with the double bond of OLAM through a radical reaction. In syntheses employing only elemental sulfur, this reaction can occur continuously due to the excess sulfur available leading to agglomerated and polydisperse particles. Within our typical synthesis, sulfur acts as a limiting reagent and the reaction cannot proceed continuously. (Scheme 5) Elemental sulfur can also react with OLAM to form an etchant shown to be capable of improving nanoparticle size and shape.

At higher temperatures (170 °C), distillation fractions indicate that OLAM reacts with both the −OCH_2_CH_3_ group lost from the LCEE-OLAM amide reaction as well as elemental sulfur, resulting in the formation of a secondary amine and sulfur bridge. The initial reaction of OLAM and the −OCH_2_CH_3_ group is seen in syntheses employing LCEE, with and without sulfur, and is indicated by the absence of the single amine peak at 1.17 ppm (Figure 5, black and red plot, respectively) seen in neat OLAM (Figure 3a, light blue plot). The presence of the ethyl is indicated by a multiplet corresponding to the additional −CH_3_ at 1.12 ppm (Figure 5, black plot). In addition, the prominent water peak in the two-sulfur synthesis at 1.52 ppm caused by the loss of water as a byproduct further supports this reaction (Figure 5, black plot). When elemental sulfur is used in conjunction with LCEE, excess sulfur may react with the double bond indicated by the quartet seen at 3.67 ppm. While the quartet is most prominent in high temperature distillation fractions and syntheses employing only elemental sulfur, low-intensity peaks in this position are also present in fractions removed at 145 and 160 °C (Figure 3a, black plot). This quartet is also seen in distillation fractions removed at 170 °C in syntheses utilizing elemental sulfur as the sole sulfur source at 3.64 ppm (Figure 5, blue plot). During distillation of a typical Ni_0.8_Co_2.2_S_4_ synthesis, this fraction yields the majority of product removed.

FTIR of the Ni_*x*_Co_3–*x*_S_4_ nanoparticles concurs with the conclusions from the NMR data, indicating the predominant presence of OLAM on the nanoparticle surface (Figure S13) regardless of the sulfur precursor(s) used. The presence of OLAM is evident by peaks indicating the amine at 1620 cm^–1^ corresponding to the N–H bending, and at 1310 cm^–1^ corresponding to the C–N stretching in OLAM.^36^ In addition, the peak at 1578 cm^–1^ indicates the NH_2_ scissoring also corresponding to OLAM.^42^ The broad peak at 3430 cm^–1^ could correspond to the N–H stretching of OLAM or to the O–H stretching vibration from water within the system or the alcohol group of ethanol.^36^ The preceding evidence suggests that the dominant ligand on the nanoparticle surface is formed by reaction of the leaving group of LCEE with OLAM and elemental sulfur.

**Scheme 4:**
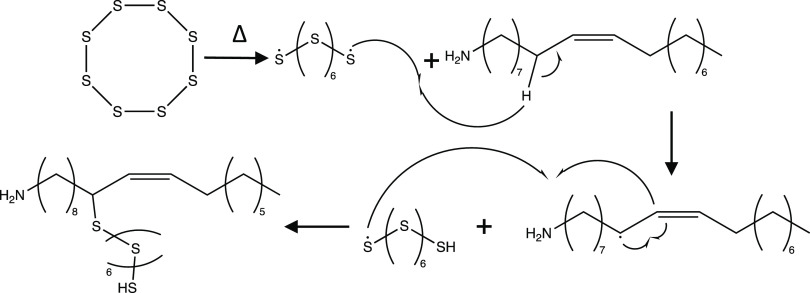
Proposed Mechanism—Radical Reaction of Sulfur and OLAM

**Scheme 5:**
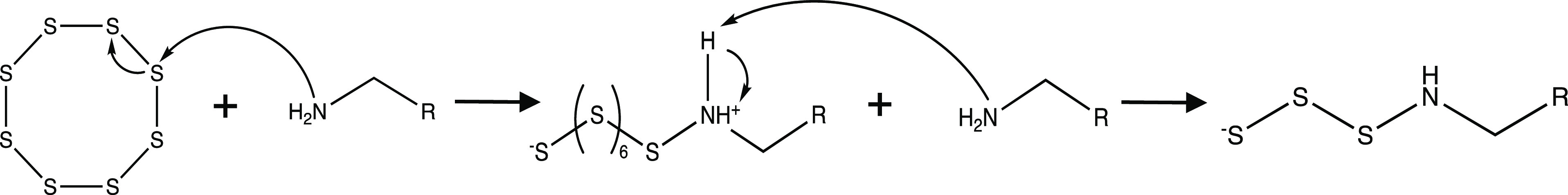
Proposed Mechanism—Formation of Sulfur and OLAM Etchant

Based on the preceding information, we propose that the formation of ligands following the thermal decomposition of metal–organic precursors and their sulfidation by H_2_S is dominated by interactions of OLAM and the sulfur precursor(s) present (Scheme 1). The presence of hydrogen sulfide within all reactions was confirmed by hydrogen sulfide test strips (WaterWorks hydrogen sulfide test strips), which indicate H_2_S through a color change. Three main types of ligands are present on the typical Ni_0.8_Co_2.2_S_4_ nanoparticle surface: (1) a thioacetal-amide formed by reaction of LCEE, OLAM, and elemental sulfur; (2) neat OLAM; and (3) OLAM with an additional ethyl group and sulfur bridge as shown by FTIR, ^1^H NMR, and ^13^C NMR. In low temperature distillations (145 °C), syntheses employing LCEE as the only sulfur source give rise to an amide formed by the condensation reaction of LCEE and OLAM. In our two-sulfur synthesis, elemental sulfur is shown to replace the oxygen of this amide forming a thioacetal with the LCEE-OLAM complex. At higher distillation temperatures, evidence indicates that excess OLAM can react with the leaving group of LCEE, -OCH_2_CH_3_ forming a secondary amine. The presence of a sulfur bridge is also prominent in fractions removed at higher distillation temperatures (over 170 °C). Following the thermal decomposition of sulfur, a radical reaction could occur between sulfur and OLAM at the double bond (Figure 5 and Scheme 4). The small quantity of sulfur employed in a typical Ni_0.8_Co_2.2_S_4_ synthesis would allow for the formation of minimal sulfur bridges between OLAM complexes. In reactions only utilizing elemental sulfur as a sulfur source, the excess sulfur would result in continuous growth causing agglomerated, polydisperse particles with high organic loading. The use of LCEE and elemental sulfur in tandem allows for limited growth of nanoparticles, improving the final size, shape, and distribution of the product. In addition, elemental sulfur may react with OLAM to form an etchant species (Figure 5 and Scheme 5). Previous work has shown this complex to also be beneficial for the size and shape of final nanoparticles.^26^

**Figure 6:**
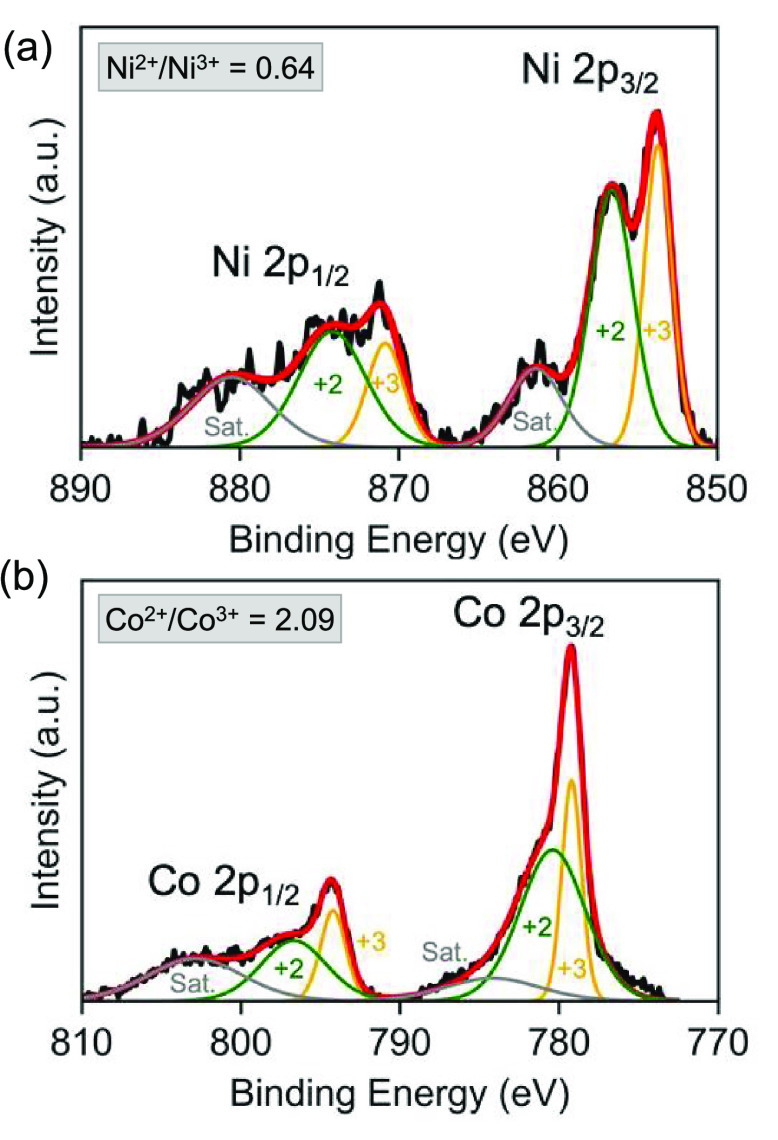
Background-subtracted XPS spectra of three random locations of a Ni_0.80_Co_2.20_S_4_ nanoparticle show mixed 2+ and 3+ oxidation states of Ni and Co cations. (a) XPS spectra of Ni 2p deconvoluted into Ni^2+^ and Ni^3+^ signals with additional satellite peaks. (b) Co 2p spectra deconvoluted into Co^2+^ and Co^3+^ signals with additional satellite peaks.

XPS measurements of the Ni_0.8_Co_2.2_S_4_ synthesis indicate that nickel and cobalt ions co-exist in the 2+ and 3+ oxidation states within the nanoparticles. The XPS spectra of Ni 2p were deconvoluted into Ni^2+^ and Ni^3+^ signals with additional satellite peaks (Figure 6a) based on the average of three scans of random locations on the nanoparticle surface (Figure S14). The peaks at 853.71 (area: 20.23%) and 870.85 eV (area: 9.30%), with a 17.14 eV separation, were assigned to Ni 2p_3/2_ and Ni 2p_1/2_ respectively, from Ni^2+^. The peaks at 856.62 (area: 27.11%) and 874.19 (area: 19.12%), with a 17.57 eV separation, are assigned to Ni 2p_3/2_ and Ni 2p_1/2_, respectively, from Ni^3+^.^49^ A Ni^2+^/Ni^3+^ ratio of 0.64 was obtained from the calculations. Likewise, the Co 2p spectra were deconvoluted into Co^2+^ and Co^3+^ signals with additional satellite peaks (Figure 6b) based on the average of three scans of random locations on the nanoparticle surface (Figure S14). The peaks at 780.40 (area: 37.14%) and 796.68 eV (area: 14.84%) were assigned to Co 2p_3/2_ and Co 2p_1/2_, respectively, corresponding to Co^2+^. The peaks at 779.21 (area: 16.23%) and 794.21 (area: 8.59%), with a 14.99 eV separation, were assigned to Co 2p_3/2_ and Co 2p_1/2_, respectively, and correspond to Co^3+^.^50^ A Co^2+^/Co^3+^ ratio of 2.09 was obtained from the calculations. The satellite peaks from Ni 2p and Co 2p spectra demonstrate the transition of an electron from a 3d orbital to the unoccupied 4s orbital in the case of the ejection of the core 2p photoelectron. XPS measurements confirm that the nickel cobalt sulfide sample contains Ni^2+^, Ni^3+^, Co^2+^, and Co^3+^, in agreement with previously reported literature.^51−54^ The mixed valence, redox-active cations commonly found in nickel cobalt sulfides and oxides have been shown to be advantageous to electrochemical performance.

### 4.3 Electrochemical Measurements

To investigate the potential application of Ni_*x*_Co_3–*x*_S_4_ nanoparticles as the active material in electrochemical batteries and supercapacitors, a composite electrode made of carbon black and ligand-removed nanoparticles was fabricated and tested in a typical half-cell. Since nanoparticles were passivated by insulating long-chain OLAM and OLAC ligands, a heat treatment process was first employed to expose the redox-active surface and enhance the conductivity of the composite material. Carbon black was employed here as a porous conductive network to facilitate nanoparticle aggregation on its surface during the heat treatment process, increasing the conductivity of the composite material. The heat-treated composite was then loaded onto a Ni foam current collector and submerged in a 6 M KOH electrolyte solution for testing.

**Figure 7:**
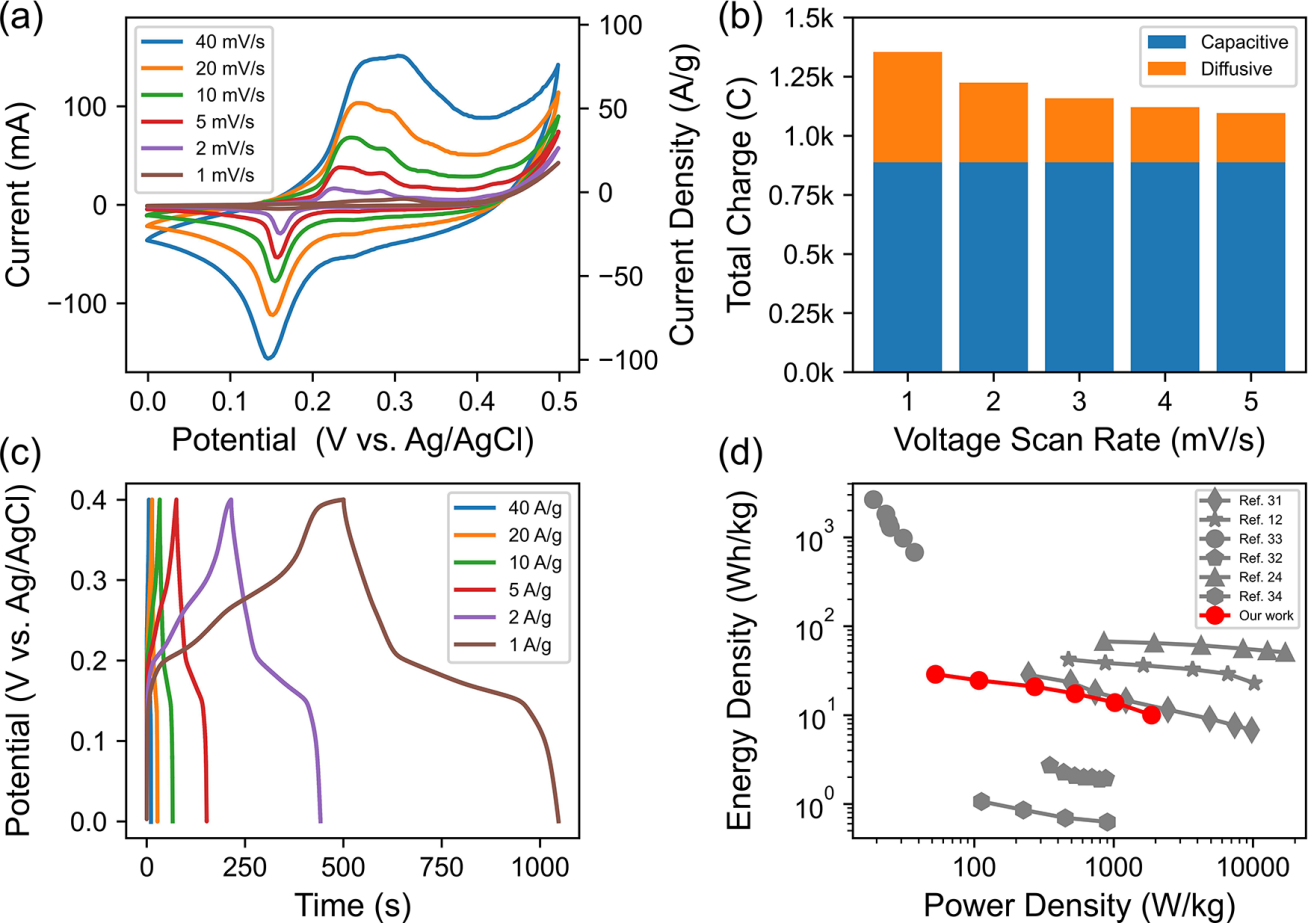
Characterization of the electrochemical performance of Ni_0.80_Co_2.20_S_4_. (a) CV plot at different scan rates indicates Faradaic behavior of the nanoparticles. (b) GCD plots at different current densities show a symmetric charge and discharge profile with a columbic efficiency of nearly 100%. (c) Total charge shows both capacitive and diffusion-controlled charge contributions, with the capacitive-controlled charge contributing the most to the total stored charge. (d) Ragone plot shows a comparison of the energy and power density of our work to previously published literature on nickel cobalt sulfides and oxides.

The shape of the CV curves of the composite electrode exhibited redox pseudocapacitive characteristics, which was expected from a nanoparticle-coated Ni foam electrode. Specifically, the CV curve (Figure 7a) between 0.2 and 0.4 V displayed two distinct redox peaks, likely corresponding to the redox potentials of nanoparticles and Ni substrate. Notably, the double-layer capacitance was not dominant even at high voltage scan rates, indicating a strong Faradaic behavior of the electrode characteristic of a battery-type electrode. To further examine whether the electrode system exhibits super capacitive charge storage behavior, we calculated the total charge stored on the electrode using CV curves at low charging and discharging speeds (low voltage scan rates) and fitted the CV curves to a semi-infinite linear diffusion model, as described by Perera et al.^55^ (Figure S15). The results (Figure 7b) revealed that the fast surface capacitive charge, which is independent of voltage scan rate, clearly dominated the total charge stored on the electrode at low voltage scan rates. This finding suggests that the Ni_*x*_Co_3–*x*_S_4_ (*x* = 0.80) nanoparticles electrode stores energy primarily through fast surface redox reactions, rather than slow ion diffusional processes. This result motivates the use of nanometer sized as active materials, as they possess a large surface to volume ratio.

The GCD curve (Figure 7c) of the Ni_*x*_Co_3–*x*_S_4_ electrode, exhibiting a symmetric charge and discharge profile with a columbic efficiency of nearly 100%. The shoulder observed at approximately 0.15 V corresponds to the Faradaic charge and discharge process that provides high energy density to the electrode. The specific capacitances *C*_sp_ of the Ni_*x*_Co_3–*x*_S_4_ electrode at various current densities are determined from the GCD curve using the equation , where  represents the current density, and Δ*t*_dis_ and Δ*V*_dis_ correspond to the discharge time and voltage change of the discharge segment of the GCD curve, respectively. At a high current density of 40 F/g, the Ni_*x*_Co_3–*x*_S_4_ electrode material exhibits a *C*_sp_ of 536 F/g, while at a low current density of 1 A/g, it demonstrates a high *C*_sp_of 1367 F/g. These results align well with previously reported values for Ni–Co–S-based supercapacitor materials in the literature, where the *C*_sp_ ranges from 1000 to 2000 F/g at 1 A/g current density.^8^ Additionally, the energy density (*E*) of the electrode is determined using the equation , and it is found to be 28.8 and 10.0 Wh/kg at 1 and 40 A/g, respectively. Furthermore, the power density (*P*) of the electrode is calculated using the formula , and it is determined to be 52.7 W/kg at 1 A/g and 1867.7 W/kg at 40 A/g, which are consistent with previous literature reports. It is important to note that the results presented in this study are preliminary, and further optimization is required to fully realize the potential of Ni_*x*_Co_3–*x*_S_4_ nanoparticles as a supercapacitive electrode material. Design parameters such as the electrolyte composition, electrode thickness, and fabrication method have not yet been fully explored, which may significantly affect the ionic resistance, impedance, and electrochemically active surface area of the electrode. Nevertheless, our findings demonstrate the potential of Ni_*x*_Co_3–*x*_S_4_ nanoparticles as a promising electrode material for energy storage applications. With further optimization, this material system may serve as a competitive alternative to the currently available supercapacitor electrode materials (Figure 7d).^12,24,31−34^

## 5 Conclusions

In summary, we have presented a cost-effective, scalable synthetic approach for the preparation of nickel cobalt sulfide nanoparticles using excess ligands, nickel(II) acetylacetonate, cobalt(II) acetylacetonate, LCEE, and sulfur. The synthesized nanoparticles were low-dispersity and sphere-like in shape with an average diameter of less than 15 nm and size dispersion below 15%. We have demonstrated that the particle size and size dispersion can be tuned by adjusting the time, temperature, and the type and ratio of excess ligands used during the synthesis. Our work provides a reliable synthetic pathway for the preparation of phase-pure nickel cobalt sulfide nanoparticles through amino acids. The presented synthetic strategy has great potential for large-scale production and practical applications in energy storage research and related fields.

## Supporting Information Available

Additional experimental details including time and temperature studies to optimize synthesis with average size and size dispersity of Ni_0.8_Co_2.2_S_4_ synthesis, TEM comparison of scalability of reaction, HAADF-STEM of single crystalline and polycrystalline mixture, compositional control study through TEM, XRD, and ICP, TEM showing impact of surfactant ratios, Scherrer analysis and calculation of XRD peaks, TGA replication of typical Ni_0.8_Co_2.2_S_4_ synthesis, NMR and FTIR spectra of all utilized precursors and syntheses described, proposed mechanisms of one sulfur source syntheses, and XPS characterization (PDF)

## References

[ref1] Theerthagiri J.; Senthil R. A.; Nithyadharseni P.; Lee S. J.; Durai G.; Kuppusami P.; Madhavan J.; Choi M. Y. Recent progress and emerging challenges of transition metal sulfides based composite electrodes for electrochemical supercapacitive energy storage. Ceram. Int. 2020, 46, 14317–14345. 10.1016/j.ceramint.2020.02.270.

[ref2] Yun Q.; Li L.; Hu Z.; Lu Q.; Chen B.; Zhang H. Layered Transition Metal Dichalcogenide-Based Nanomaterials for Electrochemical Energy Storage. Adv. Mater. 2020, 32, 1903826–1903826. 10.1002/adma.201903826. 31566269

[ref3] Huang A.; He Y.; Zhou Y.; Zhou Y.; Yang Y.; Zhang J.; Luo L.; Mao Q.; Hou D.; Yang J. A review of recent applications of porous metals and metal oxide in energy storage, sensing and catalysis. J. Mater. Sci. 2019, 54, 949–973. 10.1007/s10853-018-2961-5.

[ref4] Bretos I.; Jiménez R.; Ricote J.; Calzada M. L. Low-temperature crystallization of solution-derived metal oxide thin films assisted by chemical processes. Chem. Soc. Rev. 2018, 47, 291–308. 10.1039/c6cs00917d. 29165444

[ref5] Yu X. Y.; Lou X. W. Mixed Metal Sulfides for Electrochemical Energy Storage and Conversion. Adv. Energy Mater. 2018, 8, 1701592 10.1002/aenm.201701592.

[ref6] Rui X.; Tan H.; Yan Q. Nanostructured metal sulfides for energy storage. Nanoscale 2014, 6, 9889–9924. 10.1039/C4NR03057E. 25073046

[ref7] Chen X.; Liu Q.; Bai T.; Wang W.; He F.; Ye M. Nickel and cobalt sulfide-based nanostructured materials for electrochemical energy storage devices. Chem. Eng. J. 2021, 409, 127237–127237. 10.1016/j.cej.2020.127237.

[ref8] Xue G.; Bai T.; Wang W.; Wang S.; Ye M. Recent advances in various applications of nickel cobalt sulfide-based materials. J. Mater. Chem. A 2022, 10, 8087–8106. 10.1039/D2TA00305H.

[ref9] Zahra R.; Pervaiz E.; Yang M.; Rabi O.; Saleem Z.; Ali M.; Farrukh S. A review on nickel cobalt sulphide and their hybrids: Earth abundant, pH stable electro-catalyst for hydrogen evolution reaction. Int. J. Hydrogen Energy 2020, 45, 24518–24543. 10.1016/j.ijhydene.2020.06.236.

[ref10] Kulkarni P.; Nataraj S. K.; Balakrishna R. G.; Nagaraju D. H.; Reddy M. V. Nanostructured binary and ternary metal sulfides: Synthesis methods and their application in energy conversion and storage devices. J. Mater. Chem. A 2017, 5, 22040–22094. 10.1039/C7TA07329A.

[ref11] Huo J.; Wu J.; Zheng M.; Tu Y.; Lan Z. Flower-like nickel cobalt sulfide microspheres modified with nickel sulfide as Pt-free counter electrode for dye-sensitized solar cells. J. Power Sources 2016, 304, 266 10.1016/j.jpowsour.2015.11.062.

[ref12] Shen L.; Yu L.; Wu H. B.; Yu X. Y.; Zhang X.; Lou X. W. Formation of nickel cobalt sulfide ball-in-ball hollow spheres with enhanced electrochemical pseudocapacitive properties. Nat. Commun. 2015, 6, 6694 10.1038/ncomms7694. 25798849

[ref13] Sun M.; Tie J.; Cheng G.; Lin T.; Peng S.; Deng F.; Ye F.; Yu L. In situ growth of burl-like nickel cobalt sulfide on carbon fibers as high-performance supercapacitors. J. Mater. Chem. A 2015, 3, 1730–1736. 10.1039/C4TA04833D.

[ref14] Beka L. G.; Li X.; Liu W. Nickel Cobalt Sulfide core/shell structure on 3D Graphene for supercapacitor application. Sci. Rep. 2017, 7, 2105 10.1038/s41598-017-02309-8. 28522809 PMC5437066

[ref15] Chen Z.; Wan Z.; Yang T.; Zhao M.; Lv X.; Wang H.; Ren X.; Mei X. Preparation of Nickel Cobalt Sulfide Hollow Nanocolloids with Enhanced Electrochemical Property for Supercapacitors Application. Sci. Rep. 2016, 6, 25151 10.1038/srep25151. 27114165 PMC4844973

[ref16] Pu J.; Cui F.; Chu S.; Wang T.; Sheng E.; Wang Z. Preparation and electrochemical characterization of hollow hexagonal NiCo2S4 nanoplates as pseudocapacitor materials. ACS Sustainable Chem. Eng. 2014, 2, 809 10.1021/sc400472z.

[ref17] Xiao J.; Wan L.; Yang S.; Xiao F.; Wang S. Design Hierarchical Electrodes with Highly Conductive NiCo_2_S_4_ Nanotube Arrays Grown on Carbon Fiber Paper for High-Performance Pseudocapacitors. Nano Lett. 2014, 14, 831–838. 10.1021/nl404199v. 24437988

[ref18] Cai P.; Liu T.; Zhang L.; Cheng B.; Yu J. ZIF-67 derived nickel cobalt sulfide hollow cages for high-performance supercapacitors. Appl. Surf. Sci. 2020, 504, 144501 10.1016/j.apsusc.2019.144501.

[ref19] Lai J.; Niu W.; Luque R.; Xu G. Solvothermal synthesis of metal nanocrystals and their applications. Nano Today 2015, 10, 240–267. 10.1016/j.nantod.2015.03.001.

[ref20] Aiswarya K. M.; Raguram T.; Rajni K. S. Synthesis and characterisation of nickel cobalt sulfide nanoparticles by the solvothermal method for dye-sensitized solar cell applications. Polyhedron 2020, 176, 114267 10.1016/j.poly.2019.114267.

[ref21] Emadi H.; Salavati-Niasari M.; Sobhani A. Synthesis of some transition metal (M: 25Mn, 27Co, 28Ni, 29Cu, 30Zn, 47Ag, 48Cd) sulfide nanostructures by hydrothermal method. Adv. Colloid Interface Sci. 2017, 246, 52–74. 10.1016/j.cis.2017.06.007. 28647040

[ref22] Yu L.; Zhang L.; Wu H. B.; Lou X. W. Formation of Ni_x_Co_3–x_S_4_ Hollow Nanoprisms with Enhanced Pseudocapacitive Properties. Angew. Chem., Int. Ed. 2014, 53, 3711–3714. 10.1002/anie.201400226. 24590835

[ref23] Rajesh J. A.; Park J.-H.; Vinh Quy V. H.; Kwon J. M.; Chae J.; Kang S.-H.; Kim H.; Ahn K.-S. Rambutan-like cobalt nickel sulfide (CoNi_2_S) hierarchitecture for high-performance symmetric aqueous supercapacitors. J. Ind. Eng. Chem. 2018, 63, 73–83. 10.1016/j.jiec.2018.02.001.

[ref24] Chen W.; Zhang X.; Mo L. E.; Zhang Y.; Chen S.; Zhang X.; Hu L. NiCo_2_S_4_ quantum dots with high redox reactivity for hybrid supercapacitors. Chem. Eng. J. 2020, 388, 124109 10.1016/j.cej.2020.124109.

[ref25] Theerthagiri J.; Murthy A. P.; Lee S. J.; Karuppasamy K.; Arumugam S. R.; Yu Y.; Hanafiah M. M.; Kim H.-S.; Mittal V.; Choi M. Y. Recent progress on synthetic strategies and applications of transition metal phosphides in energy storage and conversion. Ceram. Int. 2021, 47, 4404–4425. 10.1016/j.ceramint.2020.10.098.

[ref26] Yuan B.; Tian X.; Shaw S.; Petersen R. E.; Cademartiri L. Sulfur in oleylamine as a powerful and versatile etchant for oxide, sulfide, and metal colloidal nanoparticles. Phys. Status Solidi A 2017, 214, 1600543–1600543. 10.1002/pssa.201600543.

[ref27] Thomson J. W.; Nagashima K.; MacDonald P. M.; Ozin G. A. From sulfur-amine solutions to metal sulfide nanocrystals: Peering into the oleylamine-sulfur black box. J. Am. Chem. Soc. 2011, 133, 5036–5041. 10.1021/ja1109997. 21384888

[ref28] Mourdikoudis S.; Menelaou M.; Fiuza-Maneiro N.; Zheng G.; Wei S.; Pérez-Juste J.; Polavarapu L.; Sofer Z. Oleic acid/oleylamine ligand pair: a versatile combination in the synthesis of colloidal nanoparticles. Nanoscale Horiz. 2022, 7, 941–1015. 10.1039/D2NH00111J. 35770698

[ref29] Mourdikoudis S.; Liz-Marzán L. M. Oleylamine in nanoparticle synthesis. Chem. Mater. 2013, 25, 1465–1476. 10.1021/cm4000476.

[ref30] Gao X.; Liu H.; Hidajat K.; Kawi S. Anti-Coking Ni/SiO_2_ Catalyst for Dry Reforming of Methane: Role of Oleylamine/Oleic Acid Organic Pair. ChemCatChem 2015, 7, 4188 10.1002/cctc.201500787.

[ref31] Zhu Y.; Wu Z.; Jing M.; Yang X.; Song W.; Ji X. Mesoporous NiCo_2_S_4_ nanoparticles as high-performance electrode materials for supercapacitors. J. Power Sources 2015, 273, 584 10.1016/j.jpowsour.2014.09.144.

[ref32] Shinde S. K.; Jalak M. B.; Ghodake G. S.; Maile N. C.; Yadav H. M.; Jagadale A. D.; Shahzad A.; Lee D. S.; Kadam A. A.; Fulari V. J.; et al. Flower-like NiCo_2_O_4_/NiCo_2_S_4_ electrodes on Ni mesh for higher supercapacitor applications. Ceram. Int. 2019, 45, 17192–17203. 10.1016/j.ceramint.2019.05.274.

[ref33] Kumar L.; Chauhan H.; Yadav N.; Yadav N.; Hashmi S. A.; Deka S. Faster Ion Switching NiCo_2_O_4_ Nanoparticle Electrode-Based Supercapacitor Device with High Performances and Long Cycling Stability. ACS Appl. Energy Mater. 2018, 1, 6999–7006. 10.1021/acsaem.8b01427.

[ref34] Li Z.; Wu L.; Wang L.; Gu A.; Zhou Q. Nickel cobalt sulfide nanosheets uniformly anchored on porous graphitic carbon nitride for supercapacitors with high cycling performance. Electrochim. Acta 2017, 231, 617–625. 10.1016/j.electacta.2017.02.087.

[ref35] Galanakis D.; Kourounakis A. P.; Tsiakitzis K. C.; Doulgkeris C.; Rekka E. A.; Gavalas A.; Kravaritou C.; Charitos C.; Kourounakis P. N. Synthesis and pharmacological evaluation of amide conjugates of NSAIDs with L-cysteine ethyl ester, combining potent antiinflammatory and antioxidant properties with significantly reduced gastrointestinal toxicity. Bioorg. Med. Chem. Lett. 2004, 14, 3639 10.1016/j.bmcl.2004.05.025. 15203134

[ref36] Defonsi Lestard M. E.; Díaz S. B.; Puiatti M.; Echeverría G. A.; Piro O. E.; Pierini A. B.; Altabef A. B.; Tuttolomondo M. E. Vibrational and structural behavior of l-cysteine ethyl ester hydrochloride in the solid state and in aqueous solution. J. Phys. Chem. A 2013, 117, 14243–14252. 10.1021/jp409252d. 24328050

[ref37] Williamson E. M.; Tappan B. A.; Mora-Tamez L.; Barim G.; Brutchey R. L. Statistical Multiobjective Optimization of Thiospinel CoNi_2_S_4_ Nanocrystal Synthesis via Design of Experiments. ACS Nano 2021, 15, 9422–9433. 10.1021/acsnano.1c00502. 33877801

[ref38] Zhang C.; Cai X.; Qian Y.; Jiang H.; Zhou L.; Li B.; Lai L.; Shen Z.; Huang W. Electrochemically Synthesis of Nickel Cobalt Sulfide for High-Performance Flexible Asymmetric Supercapacitors. Adv. Sci. 2018, 5, 1700375 10.1002/advs.201700375. PMC5827014 29610721

[ref39] Sperry B. M.; Kukhta N. A.; Huang Y.; Luscombe C. K. Ligand Decomposition during Nanoparticle Synthesis: Influence of Ligand Structure and Precursor Selection. Chem. Mater. 2023, 35, 570–583. 10.1021/acs.chemmater.2c03006. 36711050 PMC9879203

[ref40] Weiss I. M.; Muth C.; Drumm R.; Kirchner H. O. K. Thermal decomposition of the amino acids glycine, cysteine, aspartic acid, asparagine, glutamic acid, glutamine, arginine and histidine. BMC Biophys. 2018, 11, 2 10.1186/s13628-018-0042-4. 29449937 PMC5807855

[ref41] De Roo J.; Yazdani N.; Drijvers E.; Lauria A.; Maes J.; Owen J. S.; Van Driessche I.; Niederberger M.; Wood V.; Martins J. C.; et al. Probing Solvent–Ligand Interactions in Colloidal Nanocrystals by the NMR Line Broadening. Chem. Mater. 2018, 30, 5485–5492. 10.1021/acs.chemmater.8b02523.

[ref42] Akita C.; Kawaguchi T.; Kaneko F.; Yamamoto H.; Suzuki M. Solid-state ^13^C NMR Study on Order → Disorder Phase Transition in Oleic Acid. J. Phys. Chem. B 2004, 108, 4862–4868. 10.1021/jp037326p.

[ref43] Cara C.; Musinu A.; Mameli V.; Ardu A.; Niznansky D.; Bursik J.; Scorciapino M. A.; Manzo G.; Cannas C. Dialkylamide as both capping agent and surfactant in a direct solvothermal synthesis of magnetite and titania nanoparticles. Cryst. Growth Des. 2015, 15, 2364–2372. 10.1021/acs.cgd.5b00160.

[ref44] Calcabrini M.; Van den Eynden D.; Ribot S. S.; Pokratath R.; Llorca J.; De Roo J.; Ibáñez M. Ligand Conversion in Nanocrystal Synthesis: The Oxidation of Alkylamines to Fatty Acids by Nitrate. JACS Au 2021, 1, 1898–1903. 10.1021/jacsau.1c00349. 35574040 PMC8611721

[ref45] Baranov D.; Lynch M. J.; Curtis A. C.; Carollo A. R.; Douglass C. R.; Mateo-Tejada A. M.; Jonas D. M. Purification of Oleylamine for Materials Synthesis and Spectroscopic Diagnostics for trans Isomers. Chem. Mater. 2019, 31, 1223–1230. 10.1021/acs.chemmater.8b04198.

[ref46] Śliwiok J.; Kowalska T.; Kowalski W.; Biernat A. The influence of hydrogen-bond association on the destruction of hydroperoxides in the autoxidation process of oleyl alcohol, oleic acid, and methyl oleate. Microchem. J. 1974, 19, 362–372. 10.1016/0026-265X(74)90025-3.

[ref47] Nicolau A.; Mariath R. M.; Martini E. A.; dos Santos Martini D.; Samios D. The polymerization products of epoxidized oleic acid and epoxidized methyl oleate with cis-1,2-cyclohexanedicarboxylic anhydride and triethylamine as the initiator: Chemical structures, thermal and electrical properties. Mater. Sci. Eng., C 2010, 30, 951–962. 10.1016/j.msec.2010.04.014.

[ref48] Domazetis G.; Magee R. J.; James B. D. Tri-n-butyltin(IV) derivatives of L-cysteine ethyl ester, N-acetyl-L cysteine and α-glutamyl cysteinyl glycine (glutathione reduced). J. Organomet. Chem. 1979, 173, 357–376. 10.1016/S0022-328X(00)84791-9.

[ref49] Okabe K. L.; Sood A.; Yalon E.; Neumann C. M.; Asheghi M.; Pop E.; Goodson K. E.; Wong H. S. P. Understanding the switching mechanism of interfacial phase change memory. J. Appl. Phys. 2019, 125, 184501–184501. 10.1063/1.5093907.

[ref50] Aghavnian T.; Moussy J. B.; Stanescu D.; Belkhou R.; Jedrecy N.; Magnan H.; Ohresser P.; Arrio M. A.; Sainctavit P.; Barbier A. Determination of the cation site distribution of the spinel in multiferroic CoFe_2_O_4_/BaTiO_3_ layers by X-ray photoelectron spectroscopy. J. Electron Spectrosc. Relat. Phenom. 2015, 202, 16–21. 10.1016/j.elspec.2015.02.006.

[ref51] Dymerska A.; Kukułka W.; Biegun M.; Mijowska E. Spinel of Nickel-Cobalt Oxide with Rod-Like Architecture as Electrocatalyst for Oxygen Evolution Reaction. Materials 2020, 13, 3918 10.3390/ma13183918. 32899780 PMC7558919

[ref52] Liu T.; Liu J.; Zhang L.; Cheng B.; Yu J. Construction of nickel cobalt sulfide nanosheet arrays on carbon cloth for performance-enhanced supercapacitor. J. Mater. Sci. Technol. 2020, 47, 113–121. 10.1016/j.jmst.2019.12.027.

[ref53] Meza E.; Ortiz J.; Ruíz-León D.; Marco J. F.; Gautier J. L. Lithium-nickel cobalt oxides with spinel structure prepared at low temperature. XRD, XPS, and EIS measurements. Mater. Lett. 2012, 70, 189–192. 10.1016/j.matlet.2011.11.108.

[ref54] He G.; Qiao M.; Li W.; Lu Y.; Zhao T.; Zou R.; Li B.; Darr J. A.; Hu J.; Titirici M.-M.; et al. S, N-Co-Doped Graphene-Nickel Cobalt Sulfide Aerogel: Improved Energy Storage and Electrocatalytic Performance. Adv. Sci. 2017, 4, 1600214 10.1002/advs.201600214. PMC5238742 28105397

[ref55] Perera S. D.; Ding X.; Bhargava A.; Hovden R.; Nelson A.; Kourkoutis L. F.; Robinson R. D. Enhanced Supercapacitor Performance for Equal Co–Mn Stoichiometry in Colloidal Co_3-*x*_Mn_*x*_O_4_ Nanoparticles, in Additive-Free Electrodes. Chem. Mater. 2015, 27, 7861–7873. 10.1021/acs.chemmater.5b02106.

