# Correction to “Scalable Route to Colloidal Ni_*x*_Co_3–*x*_S_4_ Nanoparticles with Low Dispersity Using Amino Acids”

**DOI:** 10.1021/acsmaterialsau.6c00146

**Published:** 2026-10-27

**Authors:** Talisi E. Meyer, Kevin Zhijian Jiang, Ching Chun Peng, Quynh P. Sam, Minsoo Kang, Reilly P. Lynch, Jonathan L. Rowell, Judy Cha, Richard D. Robinson

In the originally published article, an error was identified in the calculation of the power densities for the Ni_*x*_Co_3–*x*_S_4_ working electrode. The correct power densities are a factor of 3.6 greater than the values originally published.

This inconsistency arises from an incorrect conversion factor used to calculate power density from the energy density. The formula for power density was given in the paper as *P* = *E*/Δ*t*. Because the units of the energy density are Wh/kg, and the power density is in W/kg, a conversion factor of 3600 s per hour must be present to convert between these two units, which was neglected during the original calculation where 1000 was used instead.

For example, the power density at 1 A/g was originally reported as 52.7 W/kg, calculated using the energy density of 28.8 Wh/kg at 1 A/g and Δ*t* = 546 s. The correct calculation should be $\frac{28.8\;Wh}{1\;kg}\times \frac{3600\;s}{1\;hr}\times \frac{1}{546\;s}$ = 189.9 W/kg at 1 A/g current density, a factor of 3.6 greater than the original reported power density.

This error is present in all reported power density values in this article specific to our work. The corrected values are provided and highlighted below. The corrected Figure 7 (specifically panel b) below replaces the one originally published in this article.

**Figure 7:**
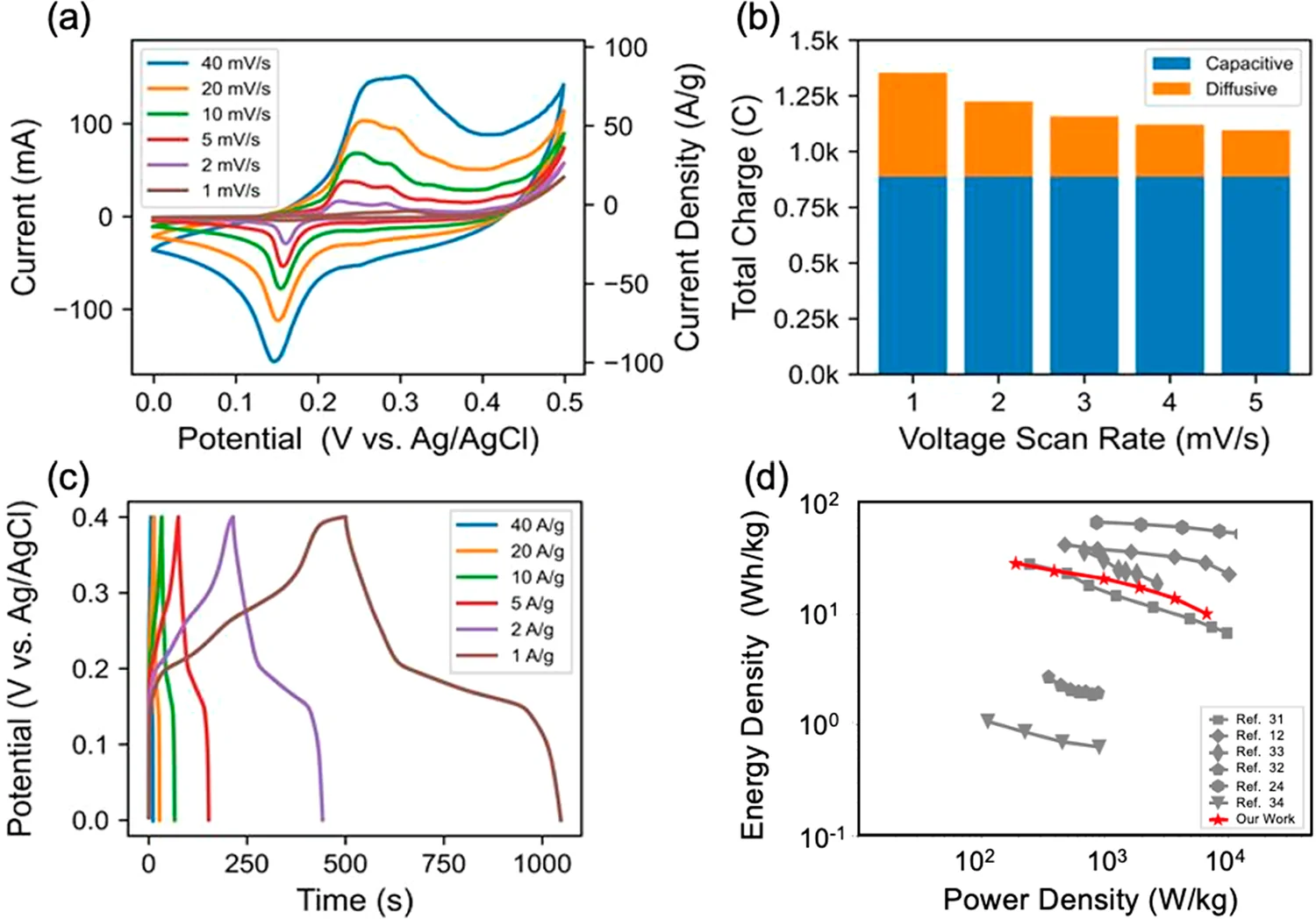
Characterization of the electrochemical performance of Ni_0.80_Co_2.20_S_4_. (a) CV plot at different scan rates indicates Faradaic behavior of the nanoparticles. **(b) Total charge shows both capacitive and diffusion-controlled charge contributions, with the capacitive-controlled charge contributing the most to the total stored charge. (c) GCD plots at different current densities show a symmetric charge and discharge profile with a Coulombic efficiency of nearly 100%.** (d) Ragone plot shows a comparison of the energy and power density of our work to previously published literature on nickel cobalt sulfides and oxides.

**Corrected Power Densities:**

52.7 W/kg at 1 A/g is corrected to 189.9 W/kg at 1 A/g.

1867.7 W/kg at 40 A/g is corrected to 6723.7 W/kg at 40 A/g.

It is important to note that this correction does not affect the conclusion of the article. Rather, it supports and further bolsters the viability of Ni_*x*_Co_3–*x*_S_4_ nanoparticles for electrochemical energy storage applications.

In addition, an error was identified in the figure references within the caption of Figure 7. In the original version, the caption states “(b) GCD plots at different current densities show a symmetric charge and discharge profile with a Coulombic efficiency of nearly 100%. (c) Total charge shows both capacitive and diffusion-controlled charge contributions, with the capacitive-controlled charge contributing the most to the total stored charge.” However, this should be corrected to indicate that panel (c) presents the GCD plots at different current densities, while panel (b) corresponds to the total stored charge. While this error was identified in the figure caption, the labels of the figure and the callouts throughout the manuscript referencing the figure are correct. The corrected figure caption is provided below with the changes in bold.

