# Supplementary material for: Scalable Route to Colloidal NixCo3–xS4 Nanoparticles with Low Dispersity Using Amino Acids
Source: ACS Mater Au. 2023 Jul 10;3(5):501–13. doi: 10.1021/acsmaterialsau.3c00016 (PMC10510506; doi:10.1021/acsmaterialsau.3c00016)
Supplement: Supplementary file 1 — mg3c00016_si_001.pdf [file mg3c00016_si_001.pdf]

Scalable Route to Colloidal $Ni_xCo_{3-x}S_4$ Nanoparticles with Low Dispersity using Amino Acids

Talisi E. Meyer[1], Kevin Zhijian Jiang[1], Ching Chun Peng[1], Quynh P. Sam[1], Minsoo Kang[1], Reilly P. Lynch[1], Jonathan L. Rowell[2], Judy Cha[1], Richard D. Robinson[1]*

[1]Department of Materials Science and Engineering, Cornell University, Ithaca, NY 14853, USA
[2]Department of Chemistry and Chemical Biology, Cornell University, Ithaca, NY 14853, USA

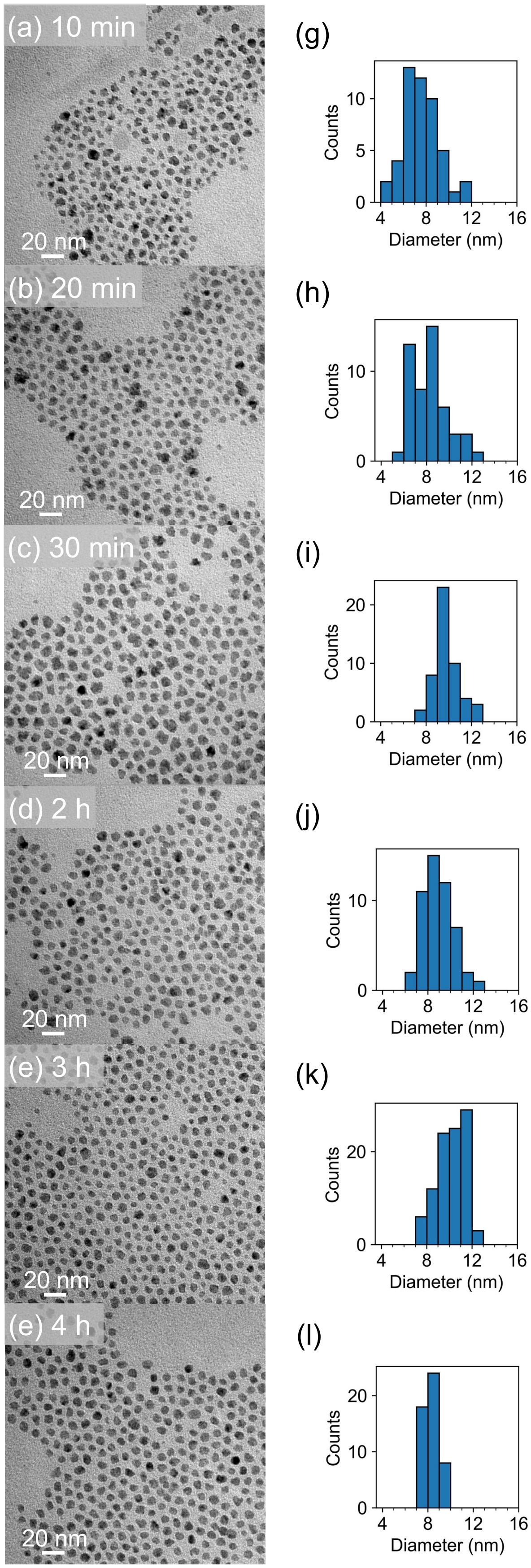

(m)

Time	Size (nm)	Size Dispersity (%)
10 min	7.5	22.1
20 min	8.2	20.0
30 min	9.8	11.6
2 h	8.9	14.8
3 h	10.1	12.4
4 h	8.3	7.7

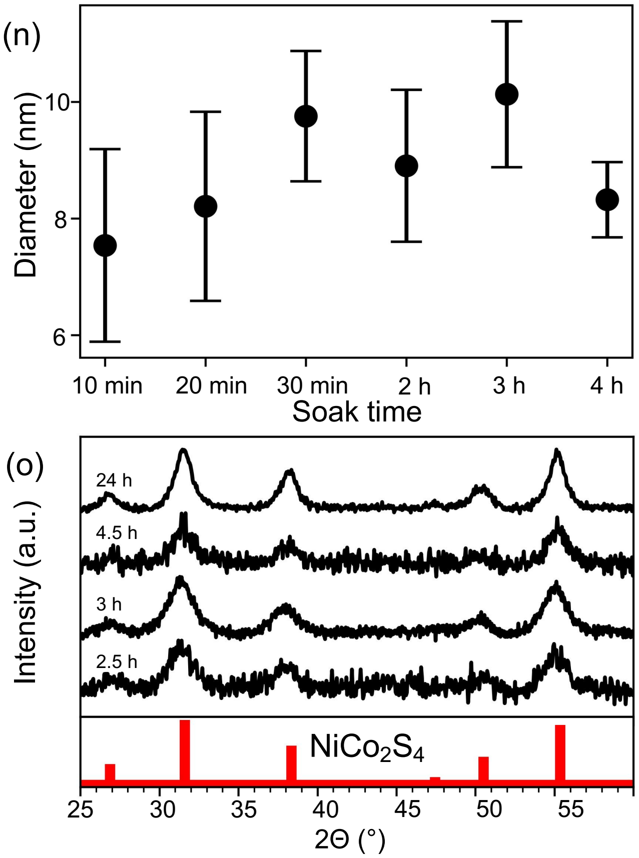

Figure S1: Nucleation and growth study of $Ni_{0.80}Co_{2.20}S_4$ nanoparticles at 170 °C soak temperature. (**a-f**) TEM Images of various time points between 0 mins and 4 hours with corresponding (**g-j**) size dispersity plots. (**m**) Table showing size in nm and size dispersity for each given time point. (**n**) Size dispersity percentage compared to the time series. (**o**) XRD of 2.5 hour, 3 hour, 4.5 hour and 24 hour samples at 170 °C compared to spinel composition. Red bars correspond to ICSD standard #624472 for $NiCo_2S_4$.

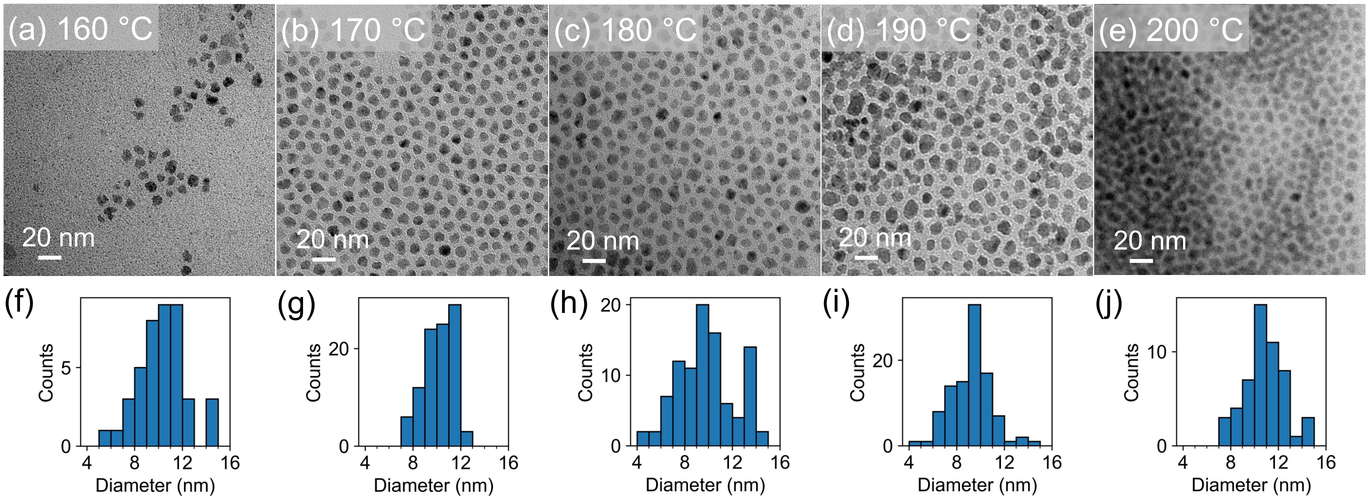

(k)

Temp. (°C)	Size (nm)	Dispersity (%)
160	10.3	18.8
170	10.1	12.4
180	10.1	25.5
190	9.3	20.1
200	10.8	15.0 + agglomerates

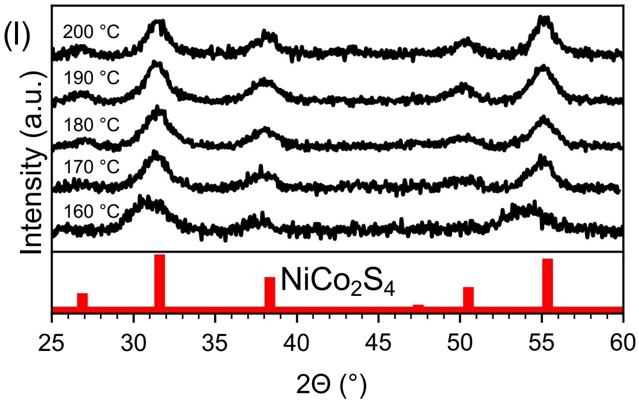

Figure S2: Temperature study for optimization of growth of $Ni_{0.80}Co_{2.20}S_4$ nanoparticles during soak portion of synthesis. All syntheses used a soak time of 3 hours. (**a-e**) TEM images of nanoparticles synthesized at 160 °C, 170 °C, 180 °C, 190 °C, and 200 °C with corresponding (**f-j**) size dispersity plots. (**k**) Table showing size in nm and size dispersity for each given temperature (**l**) XRD pattern of syntheses using various temperature each show $Ni_{0.80}Co_{2.20}S_4$ phase of nanoparticles. Red bars correspond to ICSD standard #624472 for $NiCo_2S_4$.

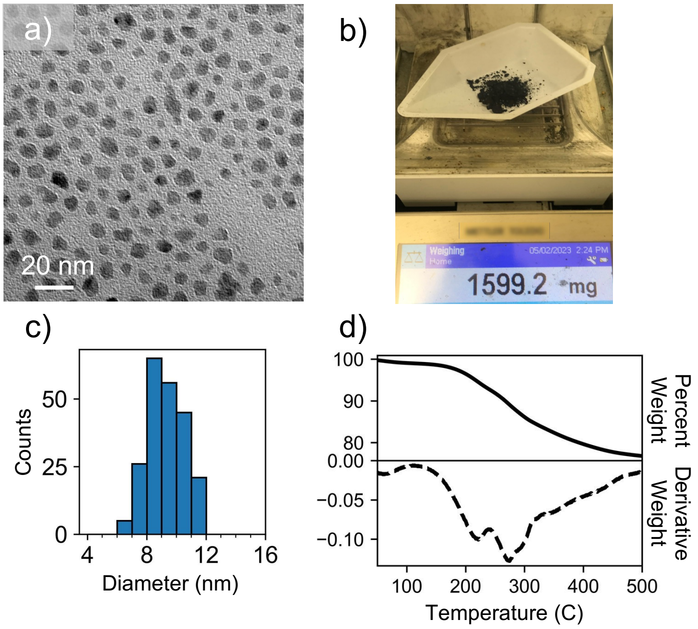

Figure S3: TEM images show the scalable nature of a typical synthesis. (a,c) The expected sphere-like shape is maintained in both reactions and the average diameter is 9.2 nm ±12.7% as demonstrated through TEM. (b) The reaction results in gram-scale yield of final products for a total mass of 1.59 g. (d) TGA shows the organic density on the nanoparticle surface is maintained compared to a typical synthesis with 23% of the final mass likely caused by ligands resulting in a yield of 1.23 g nanoparticles.

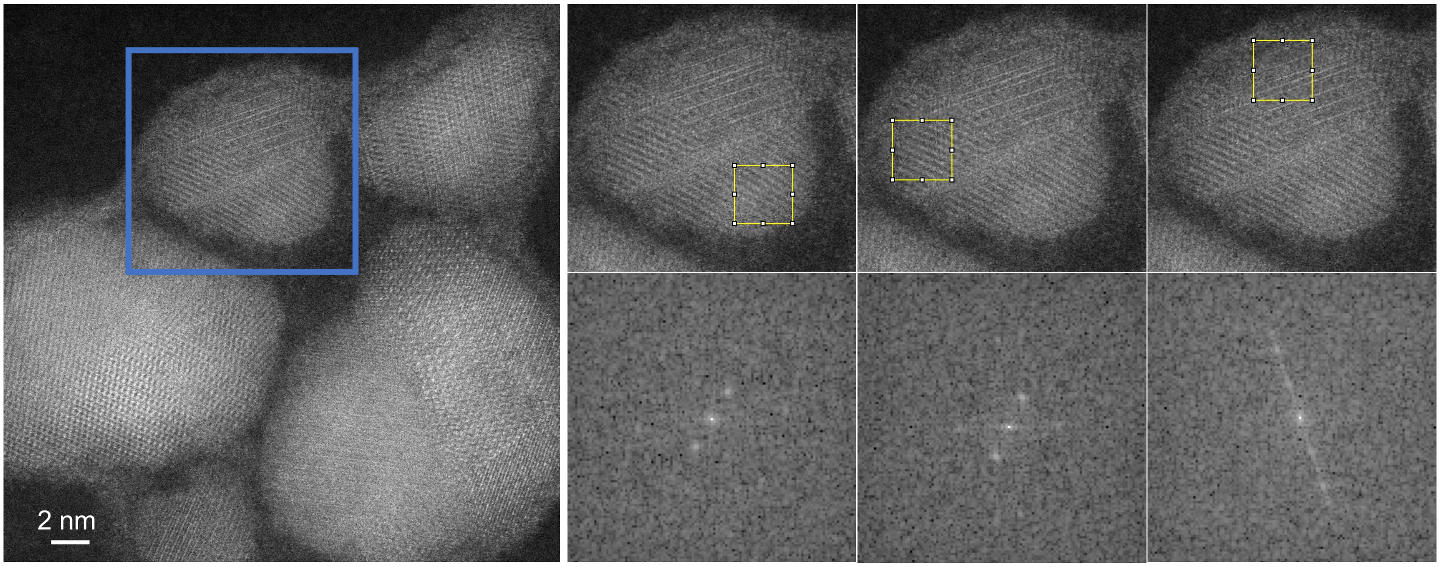

Figure S4: HAADF-STEM images of $Ni_{0.8}Co_{2.2}S_4$ nanoparticles reveal that in addition to single crystalline nanoparticles, poly-crystalline nanoparticles present (top) confirmed by the rotations of the lattice fringes in different regions of the particles, indicating grains. The nanoparticle analyzed is highlighted in blue and the specific areas of this nanoparticle analyzed by FFTs are shown in yellow.

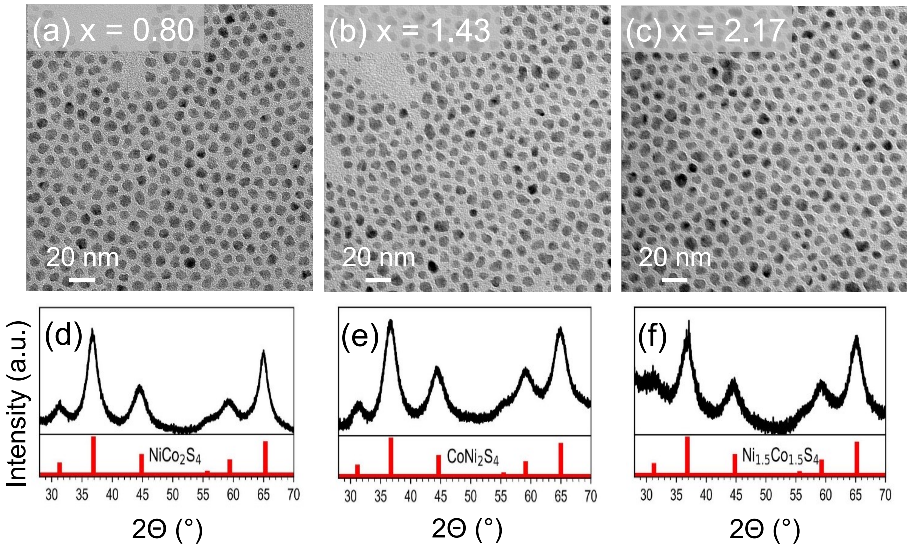

(g)

Molar ratio of precursors		Molar ratios from ICP	
Ni	Co	Ni	Co
1.0	2.0	0.80	2.20
1.5	1.5	1.43	1.57
2.0	1.0	2.17	0.83

Figure S5: Evidence of the synthetic tunability of Ni-Co cation ratios through variations of initial nickel and cobalt precursors. All syntheses were performed at 170 °C with 3 hours of soaking. (**a-c**) TEM images show no significant difference in particle morphology (**d-f**) XRD confirms structure of each synthesis. Red bars correspond to ICSD standard (**d**) #624472 for $NiCo_2S_4$, (**e**) #624473 for $Ni_{1.5}Co_{1.5}S_4$, and (**f**) #624474 for $CoNi_2S_4$. (**g**) ICP results show exact cation ratios compared to molar ratios of precursors added.

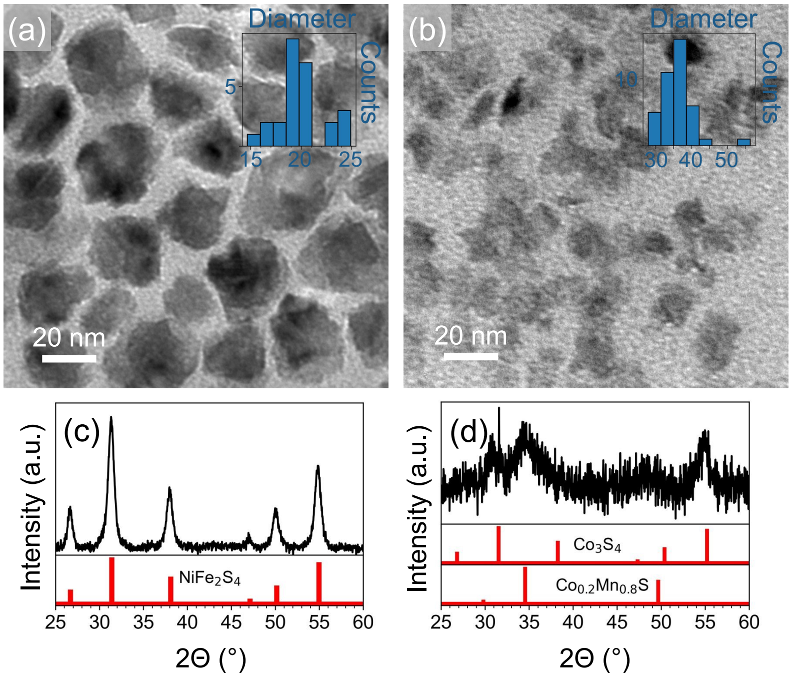

Figure S6: The versatility of our method for other ternary metal sulfides was shown through the synthesis of $NiFe_2S_4$ and $Co_{0.2}Mn_{0.8}S_4$ with excess cobalt and sulfur forming Co_3S_4. (**a,b**) TEM images show nanoparticles with (inset) size distributions of ±13.6% and ±11.5%, respectively. (**c,d**) XRD confirms the composition of nanoparticles. Red bars correspond to ICSD standard #156578 ($NiFe_2S_4$), #57435 (Co_3S_4), and #53000 ($Co_{0.2}Mn_{0.8}S_4$).

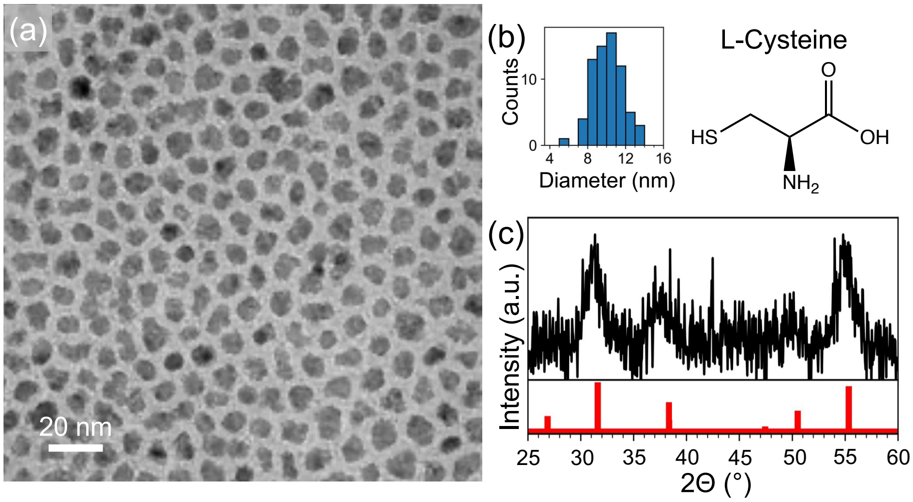

Figure S7: $Ni_{0.8}C_{2.2}S_4$ synthesis using L-Cysteine in place of LCEE. (**a**) TEM images show monodisperse particles with an average size of 10.2 nm and (**b**) size dispersion of ±17.9%. (**c**) XRD confirms thiospinel phase of final product. Red bars correspond to ICSD standard #624472 for $NiCo_2S_4$.

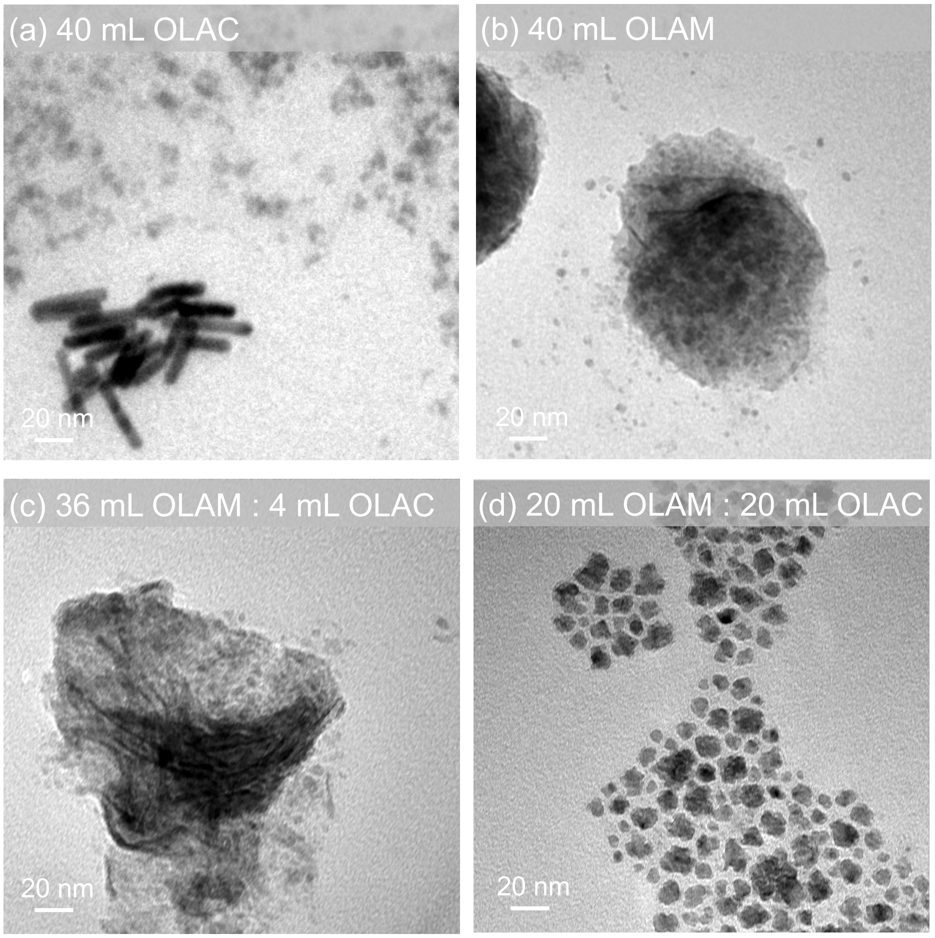

Figure S8: The impact of surfactants on the particle morphology of a typical synthesis was analyzed through TEM by varying the volume ratio of OLAM and OLAC used. All other parameters of a typical $Ni_{0.80}Co_{2.20}S_4$ synthesis were maintained. The given ratios include (**a**) 40 mL OLAC + 0 mL OLAM, (**b**) 40 mL OLAM only + 0 mL OLAC, (c) 36 mL OLAM + 4 mL OLAC, and (d) 20 mL OLAM + 20 mL OLAC.

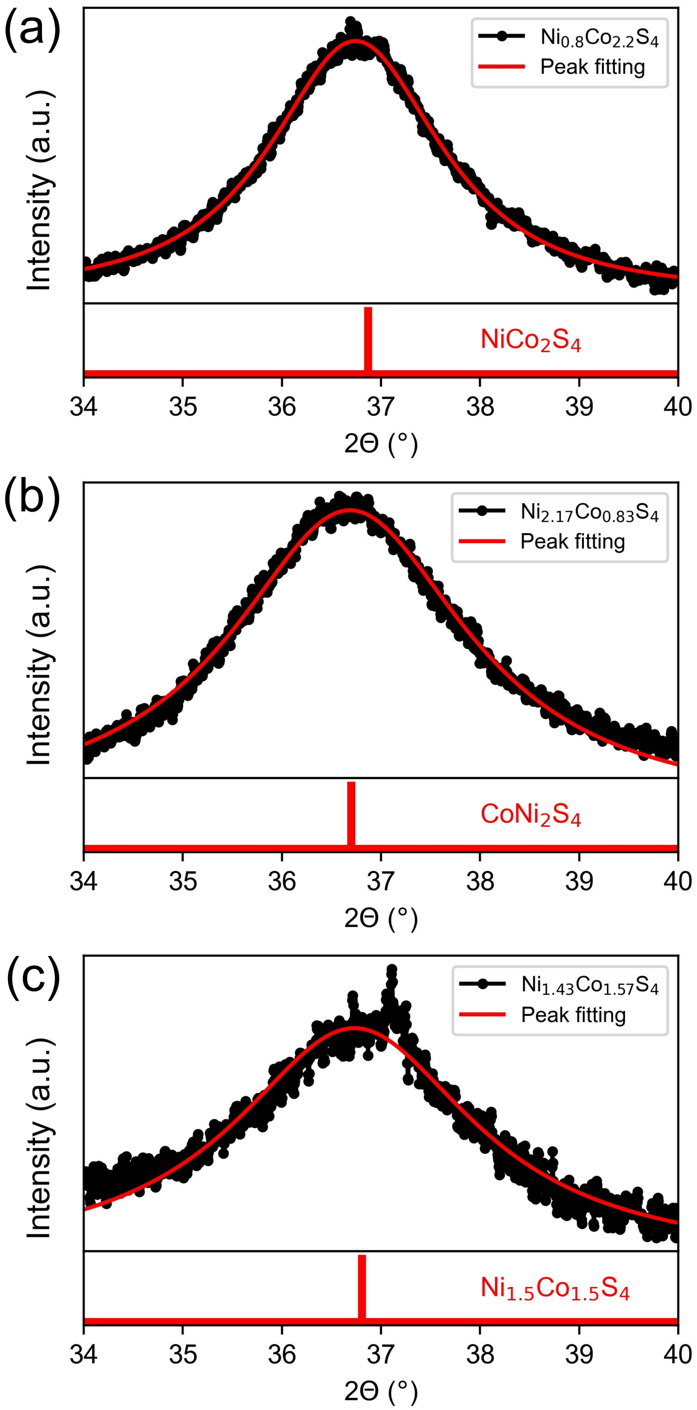

Figure S9: Gaussian fitting of the (440) reflection plane at 55° 2-theta for $Ni_xCo_{3-x}S_4$, where (**a**) x = 0.80, (**b**) x = 2.17, and (**c**) x = 1.43. The black dots are the sample signal, and the red line is the Gaussian fitting of the signal. The average crystallite sizes calculated by the Sherrer equation are 4.0 nm, 2.8 nm, and 2.8 nm, respectively (assuming the shape factor = 0.9). Red bars correspond to ICSD standard (**d**) #624472 for $NiCo_2S_4$, (**e**) #624473 for $Ni_{1.5}Co_{1.5}S_4$, and (**f**) #624474 for $CoNi_2S_4$. 119 data points and 4 variables were analyzed for each model.

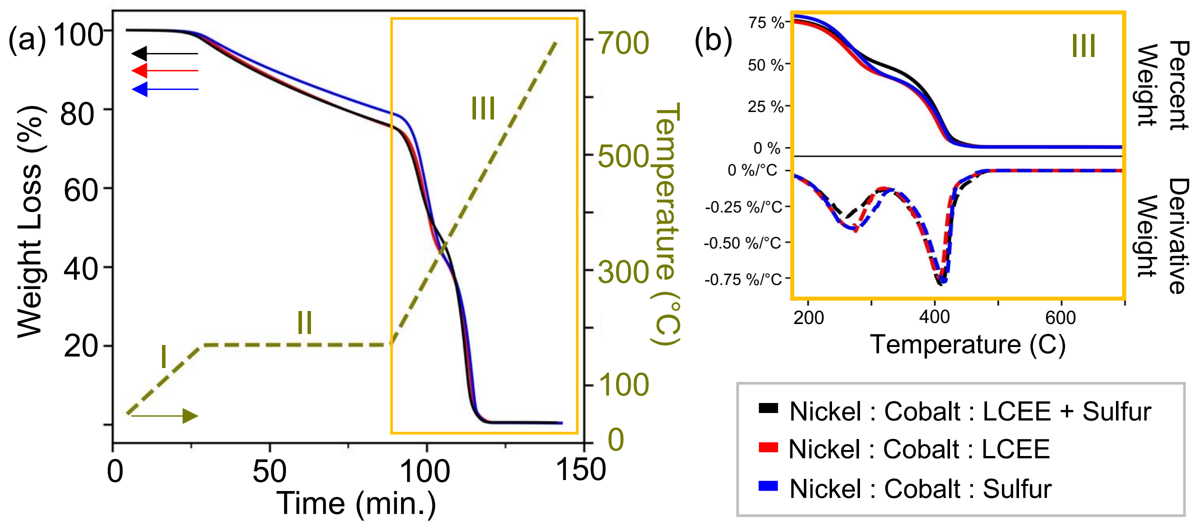

Figure S10: **(a)** Replications of a $Ni_{0.8}Co_{2.2}S_4$ synthesis through TGA using one sulfur source (LCEE or sulfur only) compared to both sulfur sources indicate that the decomposition mechanism is dependent on the sulfur source used (i.e., the initial nuclei formation depends on the sulfur precursor(s) present). Three permutations of the reaction with metal were run: LCEE only, elemental sulfur only, and LCEE plus elemental sulfur (the "$Ni_{0.8}Co_{2.2}S_4$ synthesis"). The number of moles of sulfur precursor was held constant across the three experiments (1.96 mmol of sulfur in total). The percent weight loss of all reactions was analyzed during three stages at increasing (I and III) or constant (II) temperatures and can be seen on the left y-axis corresponding to the solid black, red, and blue lines. The temperature profile is given by the right y-axis and corresponds to the olive dashed line. During the slow temperature increase (I), reactions using LCEE, with and without elemental sulfur (black and red line, respectively), exhibits less total mass loss compared to the sulfur only synthesis (blue line), evident by the percent weight loss (black, red, and blue lines and left y-axis). During the isothermal portion of the reaction (II), all precursors show a gradual decomposition. Both reactions using LCEE, with and without sulfur, show nearly identical trends in percent weight loss resulting in 25% loss of the initial mass at the end of the isothermal stage indicating similar decomposition mechanisms. The sulfur-only reaction shows a weight loss of 21% of the initial mass, suggesting a different decomposition mechanism compared to reactions employing LCEE. With increasing temperatures (III), all three reactions undergo a relatively large decrease in total percent weight between 215 °C and 240 °C which correlates with the rapid decomposition experienced by neat precursors including $Ni(acac)_2$, $Co(acac)_2$, sulfur, and OLAM + OLAC at these temperatures. Syntheses employing only one sulfur source have a larger decrease in their weight percent at this temperature compared to the two-sulfur source synthesis. The most rapid change in weight loss for all syntheses occurs above 350°C which likely corresponds to the decomposition of ligand species formed by reactions of precursors. Full decomposition of all precursors is observed above 425 °C. **(b)** Derivative TGA curves follow similar trends in the three synthesis (LCEE only, sulfur only, LCEE + S) replications indicating that above 170 °C, the remaining ligands have similar character in all reactions and that free precursors remaining post-wash can be removed through thermal decomposition at temperatures above 240°C.

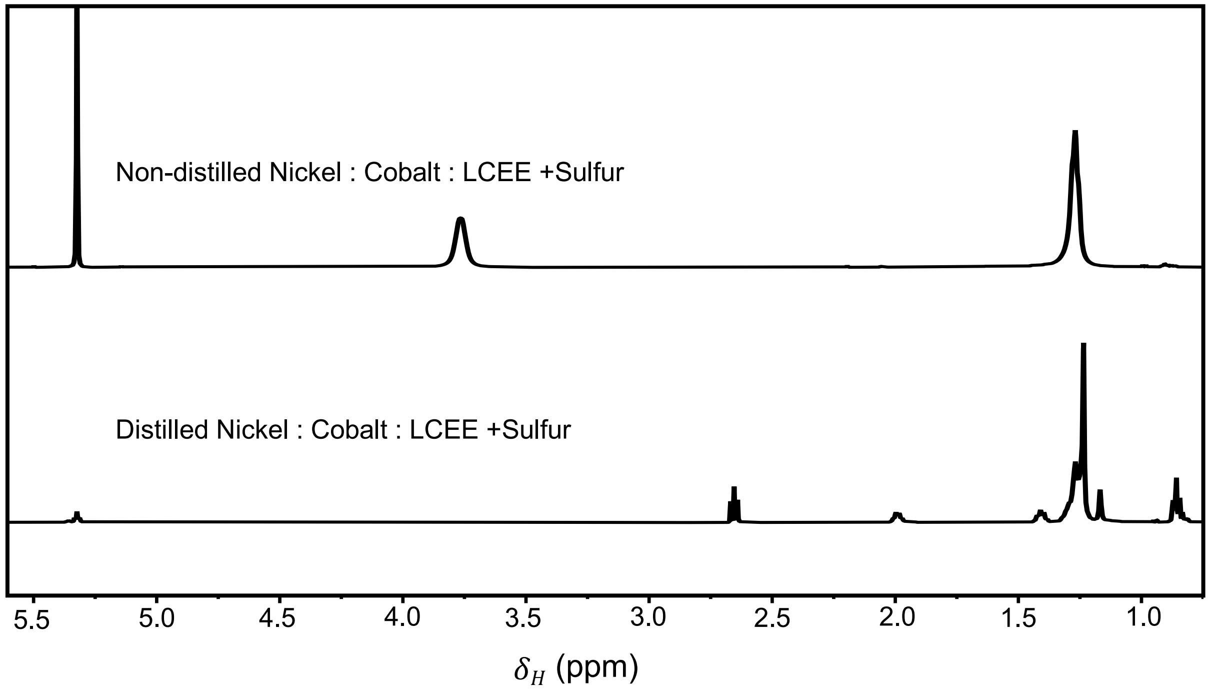

Figure S11: ^{1}H NMR spectra of non-distilled and distilled nanoparticles using LCEE and elemental sulfur. In non-distilled nanoparticles, bound ligands cause line broadening. Due to this, distilled samples allow for analysis of the characteristics of the ligands themselves.

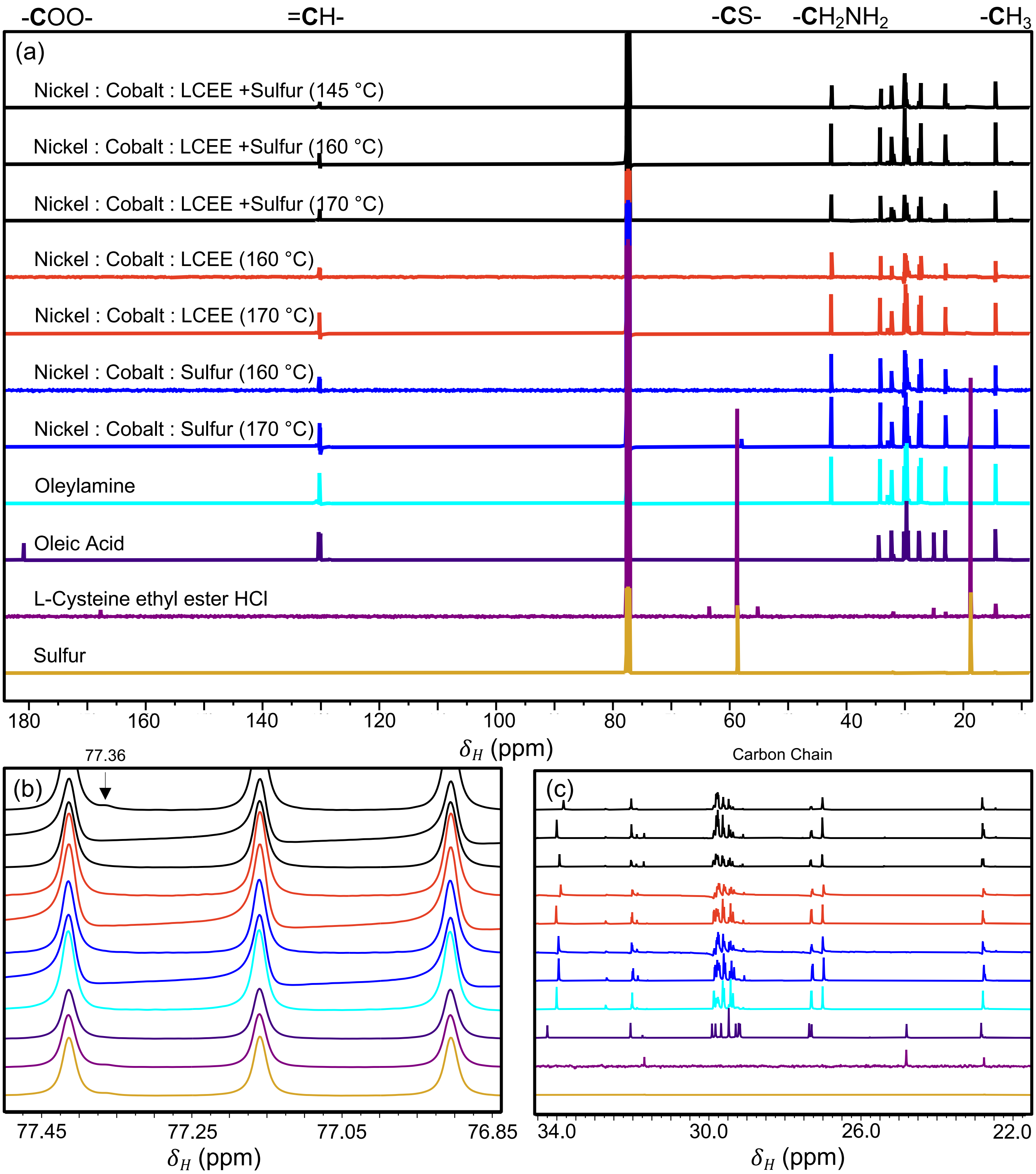

Figure S12: ^{13}C NMR spectra of distillation fractions of syntheses using one sulfur source (LCEE or sulfur only) or a typical $Ni_{0.8}Co_{2.2}S_4$ synthesis (LCEE + S) compared to neat precursors OLAM, OLAC, LCEE and sulfur. (a) Full ^{13}C NMR spectra between 10.0 ppm and 190.0 ppm shows the characteristic peaks of OLAM on the nanoparticle surface include the amine at ~42 ppm, the double bond, and the carbon chain. (b) Triplet peaks characteristic of CDCl3 show an additional peak at 77.36 ppm in distillation fractions removed at low temperatures in a $Ni_{0.8}Co_{2.2}S_4$ synthesis. This highlighted peak corresponds to the thioacetal formed by reaction of LCEE, OLAM, and elemental sulfur. (c) Magnified spectra between 22.0 ppm and 34.0 ppm show the carbon chain of the surfactants and the unique peaks of LCEE.

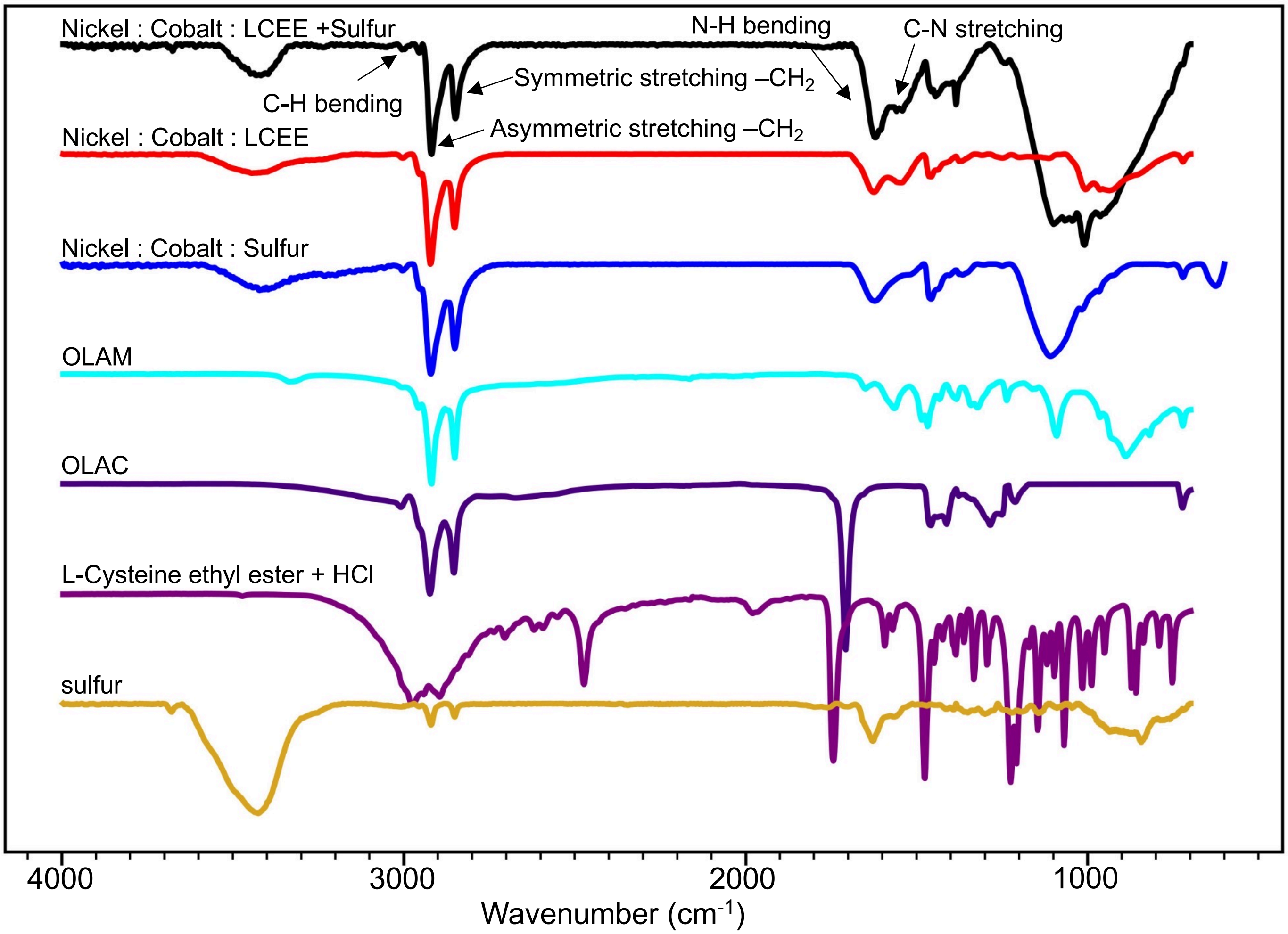

Figure S13: FTIR spectra of all synthesis components including nanoparticles from a typical $Ni_{0.8}Co_{2.2}S_4$ synthesis (LCEE + sulfur) or one sulfur source (LCEE or sulfur only) with all other parameters maintained. Final nanoparticle products are compared to neat precursors OLAM, OLAC, LCEE and sulfur. Long carbon chains, such as those in OLAM and OLAC, are evident by the asymmetric and symmetric stretching vibrations of $-CH_2$, at 2922 cm^{-1} and 2854 cm^{-1}, respectively.[37] Another indicative feature is the C-H bending corresponding to a peak at 3006 cm^{-1}.[37] The peak present at 1444 cm^{-1} further indicates the presence of an oleyl group which could correspond to OLAM or OLAC.[37] Neat oleic acid would possess characteristic peaks corresponding to the carboxylic acid at approximately 1700 cm^{-1} and 2670 cm^{-1} due to the carbonyl group and alcohol group, respectively.[43] In all distillation fractions, both peaks are not present evidence of the amine occurs at 1620 cm^{-1} corresponding to the N-H bending in OLAM, and at 1310 cm^{-1}, corresponding to the C-N stretching.[37] In addition, the peak at 1578 cm^{-1} indicates the NH_2 scissoring also corresponding to OLAM.[43] The broad peak at 3430 cm^{-1} could correspond to the N-H stretching of OLAM or to the O-H stretching vibration from water within the system or the alcohol group of ethanol.**[37]** The peaks between 1045 cm^{-1} and 1008 cm^{-1} indicate the C-O stretching modes which could be from ethanol during the washing steps.[37] In summary, the preceding evidence supports that the predominant ligand on the nanoparticle surface is OLAM either neat or following reaction with either sulfur source.

Element	Oxidation State	Orbital	1st **scan**	2nd **scan**	3rd **scan**	Average
Co 2p	Co^{2+}	Co^{2+} 3/2	37.14	34.61	32.40	34.72
		Co^{2+} 1/2	14.84	15.05	13.61	14.50
		Sum	51.98	49.66	46.01	49.22
	Co^{3+}	Co^{3+} 3/2	16.23	17.85	17.39	17.16
		Co^{3+} 1/2	8.59	9.55	9.25	9.13
		Sum	24.82	27.40	26.64	26.29
	Co^{2+}/Co^{3+}		2.09	1.81	1.73	1.88
Ni 2p	Ni^{2+}	Ni 2+ 3/2	20.23	16.83	18.6	18.55
		Ni 2+ 1/2	9.3	7.84	7.61	8.25
		Sum	29.53	24.67	26.21	26.80
	Ni^{3+}	Ni 3+ 3/2	27.11	26.15	25.75	26.34
		Ni 3+ 1/2	19.12	19.47	19.84	19.48
		Sum	46.23	45.62	45.59	45.81
	Ni^{2+}/Ni^{3+}		0.64	0.54	0.57	0.58

Figure S14: Detailed results of XPS scans of $Ni_{0.8}C_{2.2}S_4$ nanoparticles collected at three random locations on the sample. Shirley background was used for background fitting, and Gaussian profile was used for peak fitting.

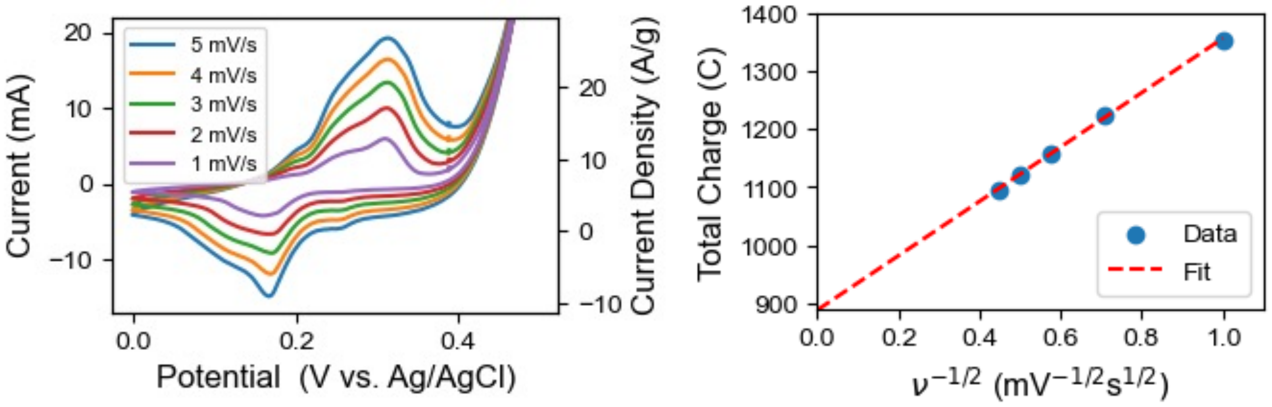

Voltage Scan Rates (mV/s)	Total Charge (C)	Diffusive Charge (C)	Capacitive Charge (C)
5	1095.7	207.8	888.0
4	1120.9	232.9	888.0
3	1158.5	270.6	888.0
2	1224.7	336.7	888.0
1	1354.0	466.1	888.0

Figure S15: Calculation of surface capacitive vs. diffusion-controlled charge storage on the electrode using the semi-infinite linear diffusion model. Only slow voltage scan rates are used for this calculation to avoid the non-linear behavior of concentration polarization on the electrode surface during CV scans.
